# Pooling of primary care electronic health record (EHR) data on Huntington’s disease (HD) and cancer: establishing comparability of two large UK databases

**DOI:** 10.1136/bmjopen-2022-070258

**Published:** 2024-02-14

**Authors:** Daniel Dedman, Rachael Williams, Krishnan Bhaskaran, Ian J Douglas

**Affiliations:** 1 Clinical Practice Research Datalink, Medicines and Healthcare Products Regulatory Agency, London, UK; 2 Epidemiology and Population Health, London School of Hygiene and Tropical Medicine, London, UK

**Keywords:** PRIMARY CARE, Health informatics, EPIDEMIOLOGY, Epidemiology

## Abstract

**Abstract:**

**Objectives:**

To explore whether UK primary care databases arising from two different software systems can be feasibly combined, by comparing rates of Huntington’s disease (HD, which is rare) and 14 common cancers in the two databases, as well as characteristics of people with these conditions.

**Design:**

Descriptive study.

**Setting:**

Primary care electronic health records from Clinical Practice Research Datalink (CPRD) GOLD and CPRD Aurum databases, with linked hospital admission and death registration data.

**Participants:**

4986 patients with HD and 1 294 819 with an incident cancer between 1990 and 2019.

**Primary and secondary outcome measures:**

Incidence and prevalence of HD by calendar period, age group and region, and annual age-standardised incidence of 14 common cancers in each database, and in a subset of ‘overlapping’ practices which contributed to both databases. Characteristics of patients with HD or incident cancer: medical history, recent prescribing, healthcare contacts and database follow-up.

**Results:**

Incidence and prevalence of HD were slightly higher in CPRD GOLD than CPRD Aurum, but with similar trends over time. Cancer incidence in the two databases differed between 1990 and 2000, but converged and was very similar thereafter. Participants in each database were most similar in terms of medical history (median standardised difference, MSD 0.03 (IQR 0.01–0.03)), recent prescribing (MSD 0.06 (0.03–0.10)) and demographics and general health variables (MSD 0.05 (0.01–0.09)). Larger differences were seen for healthcare contacts (MSD 0.27 (0.10–0.41)), and database follow-up (MSD 0.39 (0.19–0.56)).

**Conclusions:**

Differences in cancer incidence trends between 1990 and 2000 may relate to use of a practice-level data quality filter (the ‘up-to-standard’ date) in CPRD GOLD only. As well as the impact of data curation methods, differences in underlying data models can make it more challenging to define exactly equivalent clinical concepts in each database. Researchers should be aware of these potential sources of variability when planning combined database studies and interpreting results.

**STRENGTHS AND LIMITATIONS OF THIS STUDY**

- This was the most comprehensive comparison to date of these two large primary care databases which are representative of the UK population.
- We were able to compare data for a subset of the same participants with the same conditions represented in both databases. This provided assurance that case definitions for Huntington’s disease and cancers were comparable in each database.
- There was no gold-standard method with which to validate case definitions, although we were able to compare cancer incidence with national reference rates.
- Although we looked at both rare and common conditions, our findings may not generalise to other conditions.

## Introduction

 Combining data from several databases in a single study offers a number of potential benefits. First, statistical power can be increased by combining data from different sources. Second, it allows greater standardisation of many aspects of study design and implementation compared with separate studies each conducted in a single database, removing a potentially important source of variability.^1^ Third, comparing results from the individual data sources provides valuable information on generalisability of results of the pooled analysis across different populations and settings.^2 3^

The Clinical Practice Research Datalink (CPRD) GOLD database of primary care electronic health records (EHRs) was established in 1987^4 5^ and is one of the most widely used^6^,^8^ and well-validated^9^,^11^ EHR databases in observational health research. The more recently established CPRD Aurum EHR database^12 13^ is somewhat larger but has been less widely used and described,^14^ with few comparisons of the two databases published to date.^15^,^17^ The databases are similar in terms of source population and setting—around 98% of the UK population is registered with a single general practice which delivers primary care and general medical services for the publicly funded UK National Health Service (NHS). However, each database is derived from a different general practice (GP) software system employing different clinical dictionaries and coding systems, user interface and data capture methods, and data structures. These differences may introduce variability in routine data recording which should be explored and potentially accounted for in the analysis. Establishing the comparability of two data sources is a necessary first step prior to combining data.

The aim of this study was to establish the feasibility of combining UK primary care data from two CPRD databases, with a focus on examining the capture of Huntington’s disease (HD) and cancer diagnoses in the databases. These are examples of both rare and common diseases, and we also intend to conduct a future study to see whether HD is associated with a lower risk of cancer, which motivated this initial work. The specific objectives were to compare the CPRD GOLD and CPRD Aurum databases in terms of incidence and prevalence of HD; incidence of 14 common cancers; and characteristics of patients with HD or common cancers; and to explore possible reasons for any observed differences.

## Methods

### Data sources

CPRD GOLD^5^ and CPRD Aurum^13^ are databases of deidentified patient EHRs collected from participating primary care practices in the UK using either the Vision (for CPRD GOLD), or EMIS Web (for CPRD Aurum) clinical computer systems. Both databases include coded details of registration and demographic information, symptoms, diagnoses, clinical investigations and laboratory tests, prescriptions for medicines and appliances, contacts with other health services, and behavioural and social factors relevant to health and well-being. CPRD GOLD includes data from all UK regions, whereas CPRD Aurum only includes data from England and Northern Ireland. CPRD GOLD includes a derived practice-level up-to-standard (UTS) date, which indicates the point after which data are considered of adequate quality for research,^5^ whereas CPRD Aurum does not yet include any practice-level data quality indicators.

Data from patients in a subset of English practices in each database are linked to other health-related datasets, including Hospital Episode Statistics Admitted Patient Care (HES APC), and death registrations from the Office for National Statistics.^18^ HES APC is a data warehouse of all episodes of inpatient care delivered by or on behalf of NHS organisations in England.^19^ It includes coded information on diagnoses (using International Classification of Diseases 10th Revision (ICD-10)) and procedures (using the OPCS Classification of Interventions and Procedures version 4 (OPCS-4)). Death registration data include date and cause of death coded using ICD-10.^20^

A number of contributing practices switched between Vision and EMIS software during the study period. Patient EHRs (including historic records) are copied from the source to target system in this process, which results in data for patients in these practices appearing in both CPRD databases for the period up to the switch. CPRD maintains a bridging file identifying these ‘overlapping’ practices, which allows deduplication of data from affected practices, and also makes it possible to compare data from the same patients and time period represented in each database.

Analyses were conducted using the November 2020 build of the CPRD GOLD and CPRD Aurum databases, containing around 20 million and 40 million patient records, respectively, and the most contemporaneous linked data available at the time. The study period was 1 January 1990–31 December 2019, but for analyses with linked data, we restricted the study period to 1 April 1998–31 December 2019, when data collection was complete for primary care and both linked data sources.

### Study population

In each database, all male and female patients were eligible for inclusion if their records met basic patient-level data quality criteria, and they contributed at least 1 day of follow-up during the study period. Analyses involving linked data were restricted to patients eligible for linkage.

### Outcomes

Our focus was on Huntingdon’s disease (HD) as an example of a rare disease, and cancer as an example of a more common disease. These specific diseases were also chosen to inform a planned investigation into cancer risks among people with HD. Patients with HD were considered incident cases if their first ever coded diagnosis in the primary care record (the index event) occurred during the study period, and they had been registered for at least 12 months and had at least two contacts with their current practice before the index event. Patients with an HD diagnosis which did not meet the incident case definition were considered prevalent cases.

We considered 14 cancer sites, covering the 10 most common sites each for females and males based on recent English cancer registrations statistics.^21^ Cancers were identified from coded diagnoses in the primary care data, and from linked HES APC and death registration data for those analyses involving linked data. Diagnoses were classified as incident if the first ever code for that site occurred during the study period and least 12 months after the start of registration, and prevalent otherwise and excluded.

For both HD and cancer outcomes, start of follow-up was the latest of: study start; 12 months after the start of their current registration with their GP and the practice UTS date (CPRD GOLD only). End of follow-up was the earliest of: study end date; date of death or transfer out of the practice; end of data collection for the practice. Index date was the date of first diagnosis for incident cases and cohort entry for prevalent cases (HD only).

### Incidence and prevalence

HD and cancer incidence were estimated as the number of new cases arising per 100 000 person-years of follow-up, stratified by age group and gender, year or calendar period (depending on numbers) and practice region (HD only). Confidence intervals (CI) were based on a normal approximation to the Poisson distribution. Crude and directly age-standardised incidence in the CPRD GOLD and CPRD Aurum databases were compared with published national data sources.^21^ Annual point prevalence was estimated as the proportion of patients registered in the database on 1 July each year with an index event on or before that date. Prevalence was stratified by age group, gender, and practice region and CIs estimated using a normal approximation to the binomial distribution.

### Characteristics of HD and patients with cancer

Characteristics of patients in the incident HD and cancer cohorts and prevalent HD cohort were described using information from the primary care record.

Demographic details included age at index date, gender and practice region.

For general health-related variables of body mass index (BMI), blood pressure, alcohol, smoking status and drinking status only those measurements made up to 3 year before and 30 days after the index date were considered, and the measurement closest to the index date was used.

Length of available follow-up in the database was calculated from cohort entry to index date for incident cohorts; and from index date to cohort exit for incident and prevalent cohorts.

The number of healthcare contacts in terms of GP visits and referrals in the 12 months before and after the index date was estimated from consultation records and from coded clinical events. Two definitions were used for referrals: the first counted referral records only and the second additionally counted clinical events with a code indicating a referral.

The medical history was defined as having a relevant coded clinical event at any time prior to index date for common conditions including anxiety and depression, hypertension, cardiovascular disease, diabetes, chronic respiratory disease, chronic kidney disease, chronic liver disease, heavy/harmful alcohol use, and cancer (in HD cohorts only).

Treatment history variables were defined as having at least one relevant prescription record in the 12 months before index date, for selected treatments of interest: anxiety and depression treatments, antihypertensives, antidiabetic treatments. Tetrabenazine treatment (indicated for HD) in the 12 months before and after index date was defined for HD cohorts only. Drug dictionaries from both databases include information from the NHS standard Dictionary of Medicines and Devices (dm+d),^22^ and reference files from NHS Digital and NHS Business Services Authority^23 24^ were used to automatically map all prescriptions with a valid dm+d code to the relevant British National Formulary (BNF) chapter.

To assess the balance in patient characteristics between CPRD GOLD and CPRD Aurum, standardised differences were calculated^25 26^ and visualised for incident HD and cancer cohorts using Love plots.^27^ The standardised difference is commonly used to assess covariate balance between treatment groups before and after propensity score matching, with a value of less than 0.1 typically taken to indicate a negligible difference in the mean or prevalence of a covariate between the groups.

### Codelists

CPRD GOLD uses Read codes^28 29^ as its main clinical thesaurus, while CPRD Aurum uses both Read and SNOMED-CT,^30 31^ and both databases also use proprietary codes to varying degrees. For prescribing, each database uses a vendor-specific formulary, based on the NHS standard dm+d.^22^ Clinical and prescribed treatment codes are represented using a different CPRD-specific identifier in each database.

Where available, codelists were selected from the London School of Hygiene and Tropical Medicine Data Compass (LSHTM), a curated repository of reusable digital resources produced by LSHTM researchers.^32^ For some conditions, including cancer^33^ previously validated codelists were available for CPRD GOLD only. From these, comparable CPRD Aurum codelists were developed using a combination of: matching on Read codes and/or text terms; Read to SNOMED-CT mapping files from NHS Digital^34^; keyword searches on the CPRD Aurum dictionary using list of keywords or ‘term sets’^35^ generated from the CPRD GOLD codelist; and a final manual review of candidate mappings by at least two experienced researchers.

Final codelists are included as separate files (see Codelists.zip in online supplemental file), and further details are provided in online supplemental file.

### Overlapping practices

Incidence and prevalence analyses were repeated in the subset of overlapping practices. To assess the impact of the GOLD UTS date on incidence rates, the analysis was repeated using follow-up time calculated both with and without applying the CPRD GOLD UTS date to both databases.

To assess the concordance of HD recording in the two databases, the practice-level bridging file was used to identify pairs of overlapping practices, and within each pair we attempted to match each patient with HD to a record in the other practice using year of birth and gender.

### Patient and public involvement

There was no patient or public involvement in the design, analysis or reporting of this study.

### Reporting

We followed the Strengthening the Reporting of Observational Studies in Epidemiology (STROBE) guidelines for reporting cross-sectional observational studies.^36^

## Results

### HD incidence and prevalence

**Figure 1:**
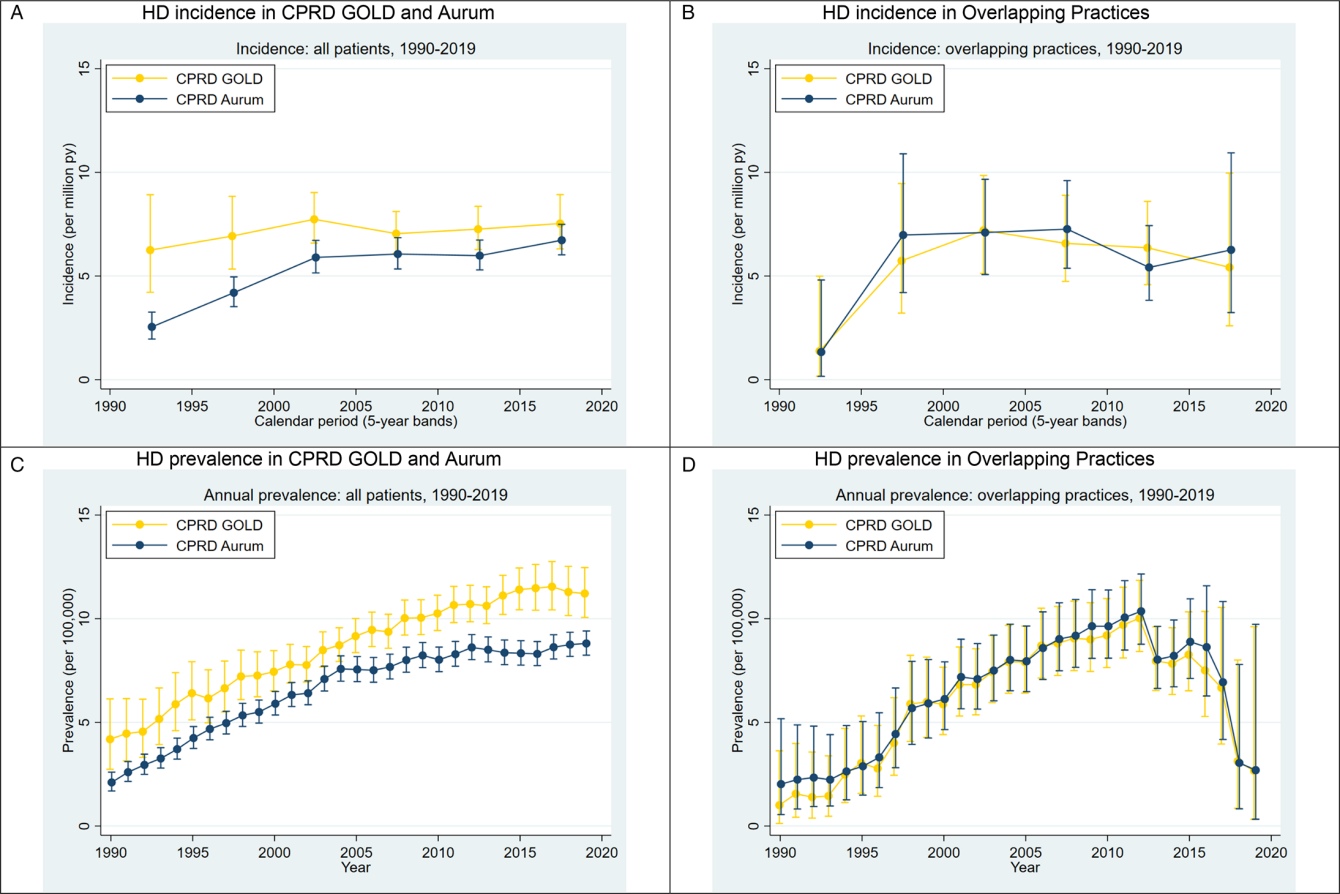
Huntington’s disease (HD) incidence and prevalence, 1990–2019: CPRD GOLD and CPRD Aurum. CPRD, Clinical Practice Research Datalink.

During the study period, there were 1948 HD cases in CPRD GOLD and 3038 in CPRD Aurum, with 40% and 42%, respectively, classified as incident (table 1). Trends in HD incidence and prevalence are shown in figure 1. In both databases, recorded incidence increased between 1990 and 2000, particularly in CPRD Aurum, and was relatively stable thereafter (figure 1A). Prevalence increased relatively steadily between 1990 and 2010 in both databases but started to level off thereafter (figure 1C). Recorded incidence and prevalence were higher in CPRD GOLD than in CPRD Aurum throughout the study period, and this trend was also apparent in all age groups and in almost all regions (online supplemental figure S1).

**Table 1:** Characteristics of incident and prevalent Huntington’s disease patients in CPRD GOLD and CPRD Aurum

	Incident cases			Prevalent cases		
	CPRD Aurum	CPRD GOLD	std.diff	CPRD Aurum	CPRD GOLD	std.diff*
Total patients (n)	**1287**	**774**		**1751**	**1174**	
Age at index date (mean (SD))	53.2 (15.8)	53.0 (15.9)	0.01	52.3 (14.7)	51.7 (14.5)	0.05
Female (n (%))	657 (51.0)	415 (53.6)	0.05	900 (51.4)	593 (50.5)	0.02
BMI† (mean (SD))	24.9 (5.4)	24.5 (4.8)	0.07	23.5 (4.4)	24.0 (4.7)	0.09
Missing BMI (n (%))	813 (63.2)	486 (62.8)	0.01	1458 (83.3)	919 (78.3)	0.13
Diastolic blood pressure (BP)† mm Hg (mean (SD))	77.1 (9.9)	76.3 (10.4)	0.08	76.7 (10.9)	77.2 (11.1)	0.04
Missing diastolic BP (n (%))	955 (74.2)	560 (72.4)	0.04	1394 (79.6)	895 (76.2)	0.08
Systolic BP† mm Hg (mean (SD))	127.0 (17.6)	125.7 (16.2)	0.08	125.4 (18.6)	126.2 (20.3)	0.04
Missing systolic BP (n (%))	955 (74.2)	560 (72.4)	0.04	1394 (79.6)	895 (76.2)	0.08
Smoking†: current/ex (n (%))	775 (63.8)	435 (59.4)	0.09	915 (59.5)	562 (54.8)	0.09
Missing smoking status (n (%))	73 (5.7)	42 (5.4)	0.01	212 (12.1)	149 (12.7)	0.02
Alcohol status[Table-fn T1_FN2]: current/ex (n (%))	836 (80.2)	565 (87.5)	0.2	749 (62.5)	572 (70.8)	0.18
Missing alcohol status (n (%))	245 (19.0)	128 (16.5)	0.07	553 (31.6)	366 (31.2)	0.01
Years of follow-up prior to index date (mean (SD))	11.3 (8.1)	7.4 (5.7)	0.55	0.4 (1.7)	0.2 (1.1)	0.14
Years of follow-up after index date (mean (SD))	5.8 (5.0)	5.6 (4.4)	0.05	5.0 (5.3)	4.9 (4.6)	0.03
Healthcare contacts						
GP visits in 12 months before index date (n (SD))	7.7 (8.4)	10.9 (10.5)	0.34	9.4 (9.8)	12.8 (12.1)	0.31
GP visits in 12 months after index date n (SD))	8.1 (8.3)	12.4 (10.9)	0.44	7.3 (8.7)	10.8 (11.1)	0.35
Referrals (definition 1) in 12 months before index date (n (SD))	0.6 (0.9)	0.9 (1.3)	0.3	0.5 (1.0)	0.7 (1.2)	0.2
Referrals (definition 1) in 12 months after index date (n (SD))	1.0 (1.3)	1.1 (1.4)	0.04	1.0 (1.6)	0.9 (1.4)	0.06
Referrals (definition 2) in 12 months before index date (n (SD))	0.5 (1.0)	0.7 (1.3)	0.21	0.3 (0.7)	0.5 (0.9)	0.19
Referrals (definition 2) in 12 months after index date (n (SD))	0.9 (1.5)	0.9 (1.4)	0.01	0.6 (1.2)	0.6 (1.1)	0.01
Medical history						
Anxiety and depression (n (%))	561 (43.6)	330 (42.6)	0.02	698 (39.9)	499 (42.5)	0.05
Hypertension (n (%))	188 (14.6)	135 (17.4)	0.08	125 (7.1)	94 (8.0)	0.03
Cardiovascular disease (n (%))	79 (6.1)	61 (7.9)	0.07	93 (5.3)	53 (4.5)	0.04
Diabetes (n (%))	44 (3.4)	26 (3.4)	<0.01	73 (4.2)	39 (3.3)	0.04
Chronic respiratory disease (n (%))	172 (13.4)	105 (13.6)	0.01	190 (10.9)	94 (8.0)	0.1
Chronic kidney disease (n (%))	28 (2.2)	22 (2.8)	0.04	38 (2.2)	14 (1.2)	0.08
Chronic liver disease (n (%))	7 (0.5)	2 (0.3)	0.05	6 (0.3)	5 (0.4)	0.01
Alcohol: heavy or harmful use (n (%))	71 (5.5)	37 (4.8)	0.03	115 (6.6)	71 (6.0)	0.02
Cancer (excluding non-melanoma skin cancer) (n (%))	73 (5.7)	34 (4.4)	0.06	64 (3.7)	34 (2.9)	0.04
**Treatment history: prescriptions in 12 months prior to index date**						
By BNF chapter						
00: Not classifiable (n (%))	226 (17.6)	22 (2.8)	0.5	661 (37.7)	107 (9.1)	0.72
01: Gastrointestinal system (n (%))	284 (22.1)	198 (25.6)	0.08	666 (38.0)	429 (36.5)	0.03
02: Cardiovascular system (n (%))	301 (23.4)	200 (25.8)	0.06	309 (17.6)	234 (19.9)	0.06
03: Respiratory system (n (%))	191 (14.8)	120 (15.5)	0.02	302 (17.2)	190 (16.2)	0.03
04: Central nervous system (n (%))	738 (57.3)	466 (60.2)	0.06	1277 (72.9)	900 (76.7)	0.09
05: Infections (n (%))	340 (26.4)	228 (29.5)	0.07	565 (32.3)	432 (36.8)	0.1
06: Endocrine system (n (%))	225 (17.5)	135 (17.4)	<0.01	245 (14.0)	152 (12.9)	0.03
07: Obstetrics, gynaecology and urinary tract disorders (n (%))	153 (11.9)	95 (12.3)	0.01	198 (11.3)	127 (10.8)	0.02
08: Malignant disease and immunosuppression (n (%))	4 (0.3)	5 (0.6)	0.05	12 (0.7)	6 (0.5)	0.02
09: Nutrition and blood (n (%))	165 (12.8)	133 (17.2)	0.12	486 (27.8)	448 (38.2)	0.22
10: Musculoskeletal and joint diseases (n (%))	225 (17.5)	148 (19.1)	0.04	276 (15.8)	203 (17.3)	0.04
11: Eye (n (%))	72 (5.6)	60 (7.8)	0.09	157 (9.0)	88 (7.5)	0.05
12: Ear, nose and oropharynx (n (%))	121 (9.4)	76 (9.8)	0.01	177 (10.1)	132 (11.2)	0.04
13: Skin (n (%))	221 (17.2)	159 (20.5)	0.09	567 (32.4)	352 (30.0)	0.05
14: Immunological products and vaccines (n (%))	105 (8.2)	41 (5.3)	0.11	261 (14.9)	105 (8.9)	0.18
15: Anaesthesia (n (%))	15 (1.2)	3 (0.4)	0.09	33 (1.9)	24 (2.0)	0.01
99: Other preparations, dressings, appliances (n (%))	121 (9.4)	80 (10.3)	0.03	435 (24.8)	256 (21.8)	0.07
Any prescription (n (%))	1062 (82.5)	660 (85.3)	0.07	1486 (84.9)	1056 (89.9)	0.15
Total prescriptions in 12 months before index date (mean (SD))	28.7 (47.3)	27.9 (38.7)	0.02	61.3 (65.5)	52.0 (52.9)	0.16
Other treatments						
Anxiety and depression treatments (n (%))	499 (38.8)	310 (40.1)	0.03	887 (50.7)	634 (54.0)	0.07
Antihypertensives (n (%))	149 (11.6)	112 (14.5)	0.09	125 (7.1)	101 (8.6)	0.05
Antidiabetic treatment (n (%))	30 (2.3)	17 (2.2)	0.01	51 (2.9)	24 (2.0)	0.06
Tetrabenazine (n (%))	65 (5.1)	51 (6.6)	0.07	261 (14.9)	172 (14.7)	0.01
Treatment in 12 months after index date						
Tetrabenazine (n (%))	156 (12.1)	98 (12.7)	0.02	251 (14.3)	189 (16.1)	0.05

* Standardised difference.

† Measurement up to 3 years prior to index date.

BMI, body mass index; BNF, British National Formulary; CPRD, Clinical Practice Research Datalink; GP, general practice; SD, standard deviation.

In the subset of overlapping practices, HD incidence (figure 1B) and prevalence (figure 1D) were very similar in both databases. In the overlapping practices, 408 of 427 (95.6%) HD cases in CPRD GOLD and 408 of 447 (91.3%) of cases in CPRD Aurum were presumptively linked on gender and year birth. The databases showed very good agreement classifying presumptively linked cases as incident vs prevalent: only 8 of 408 (1.9%) cases were discordant, with all 8 classified as prevalent in CPRD GOLD but incident in Aurum (kappa=0.96 (95% CI 0.93 to 0.99)). The small numbers of HD cases which could not be linked on gender and age were mostly from practices which split or merged with another practice after the switch from Vision to EMIS software.

### Cancer incidence

**Figure 2:**
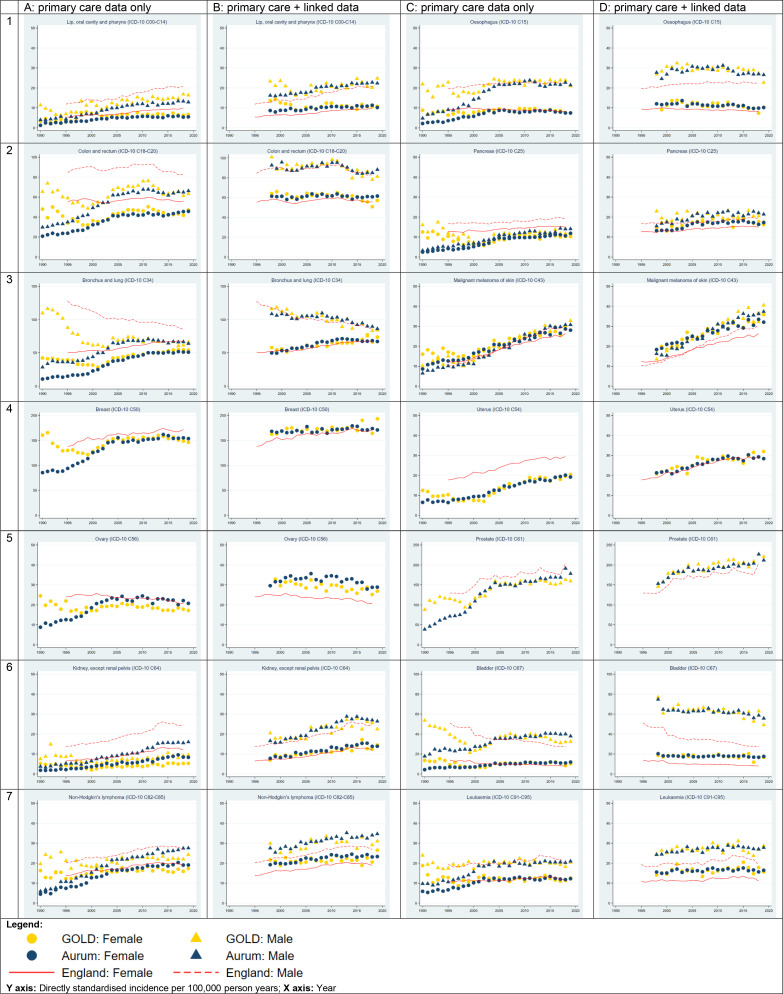
Age-standardised incidence of 14 common cancers, 1990–2019: CPRD GOLD and CPRD Aurum primary care data only (columns A and C), and with linked hospital admission (HES APC) and death registrations (ONS) data (columns B and D). Reference rates (red solid and dashed lines) are National Cancer Registration statistics for England. CPRD, Clinical Practice Research Datalink; HES APC, Hospital Episode Statistics Admitted Patient Care; ICD, International Classification of Diseases 10th Revision; ONS, Office for National Statistics.

Figure 2 compares age adjusted incidence estimated from primary care data alone (figure 2: columns A** and C**) and from linked primary care, hospitalisation and death registration data (figure 2: columns B** and D**)for the 14 cancer sites. For most cancers, incidence based on primary care data alone showed different trends in the two databases between 1990 and 2000, with incidence steady or decreasing in CPRD GOLD but increasing in CPRD Aurum. From 2000 onwards, the level and trend in incidence rates were very similar in each database. The main exceptions were ovarian (figure 2: C5) and kidney cancer (figure 2: C6), where incidence in CPRD Aurum was slightly lower than CPRD GOLD between 1990 and 2000, but higher thereafter.

### Impact of UTS date in overlapping practices

**Figure 3:**
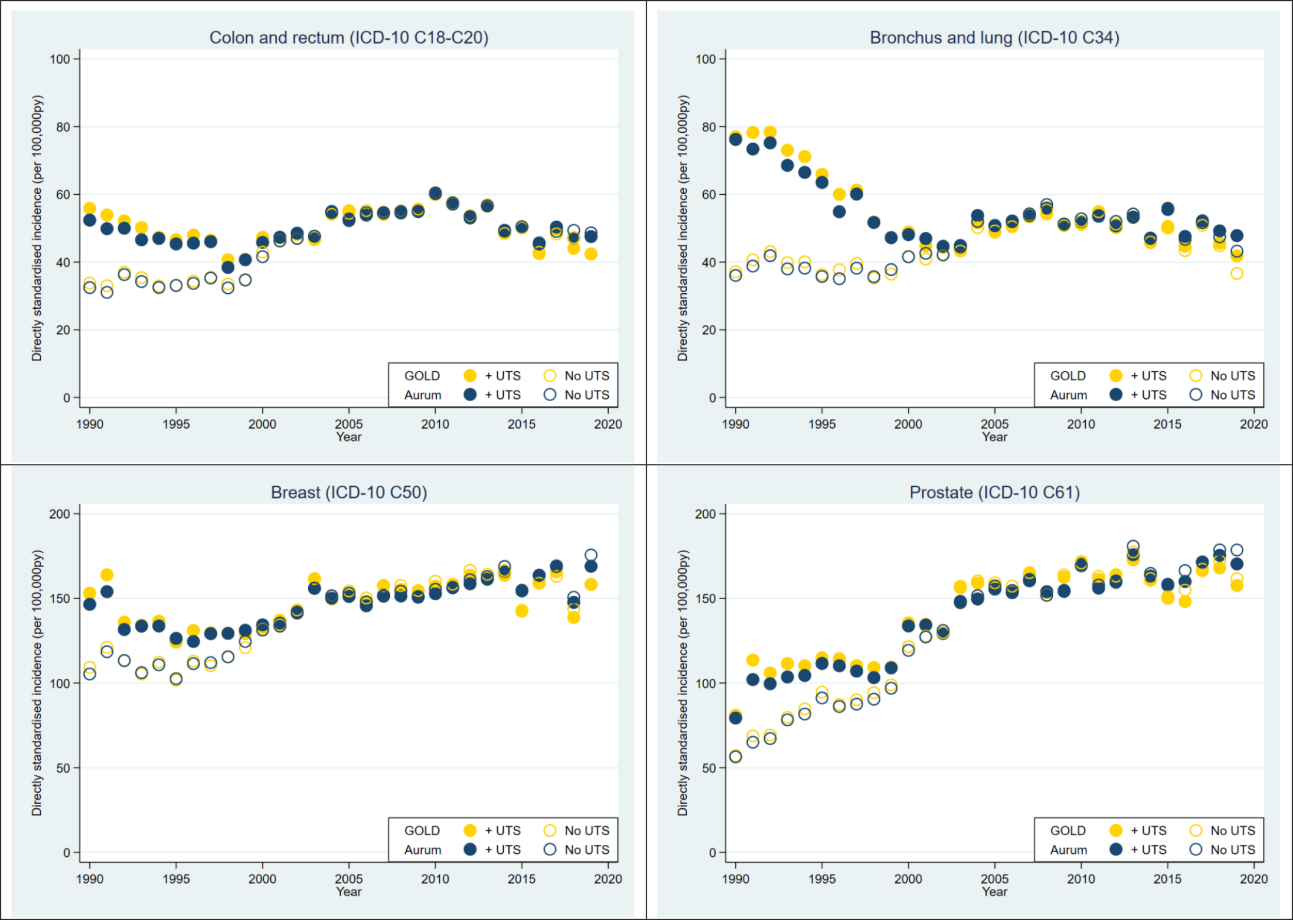
Impact of practice up-to-standard date (UTS) on age-standardised and sex-standardised incidence of four most common cancers in a subset of overlapping practices, 1990–2019: CPRD GOLD (yellow symbols) and CPRD Aurum (blue symbols). Incidence was calculated in two ways: applying the UTS filter to exclude data prior to UTS date (‘+UTS’, filled symbols), ignoring the UTS filter and including data prior to UTS date (‘no UTS’; unfilled symbols). CPRD, Clinical Practice Research Datalink; ICD-10, International Classification of Diseases 10th Revision.

Cancer incidence was estimated in overlapping practices in each database and repeated using two definitions for start of follow-up: one including and one excluding all person time prior to the CPRD GOLD UTS for each practice. Results for the four most common cancers are shown in figure 3. The effect of excluding person time prior to the CPRD GOLD UTS date was to increase recorded incidence between 1990 and 2000, with very little impact on rates thereafter. A similar effect was seen on HD incidence and prevalence (not shown).

### Characteristics of patients with HD

**Figure 4:**
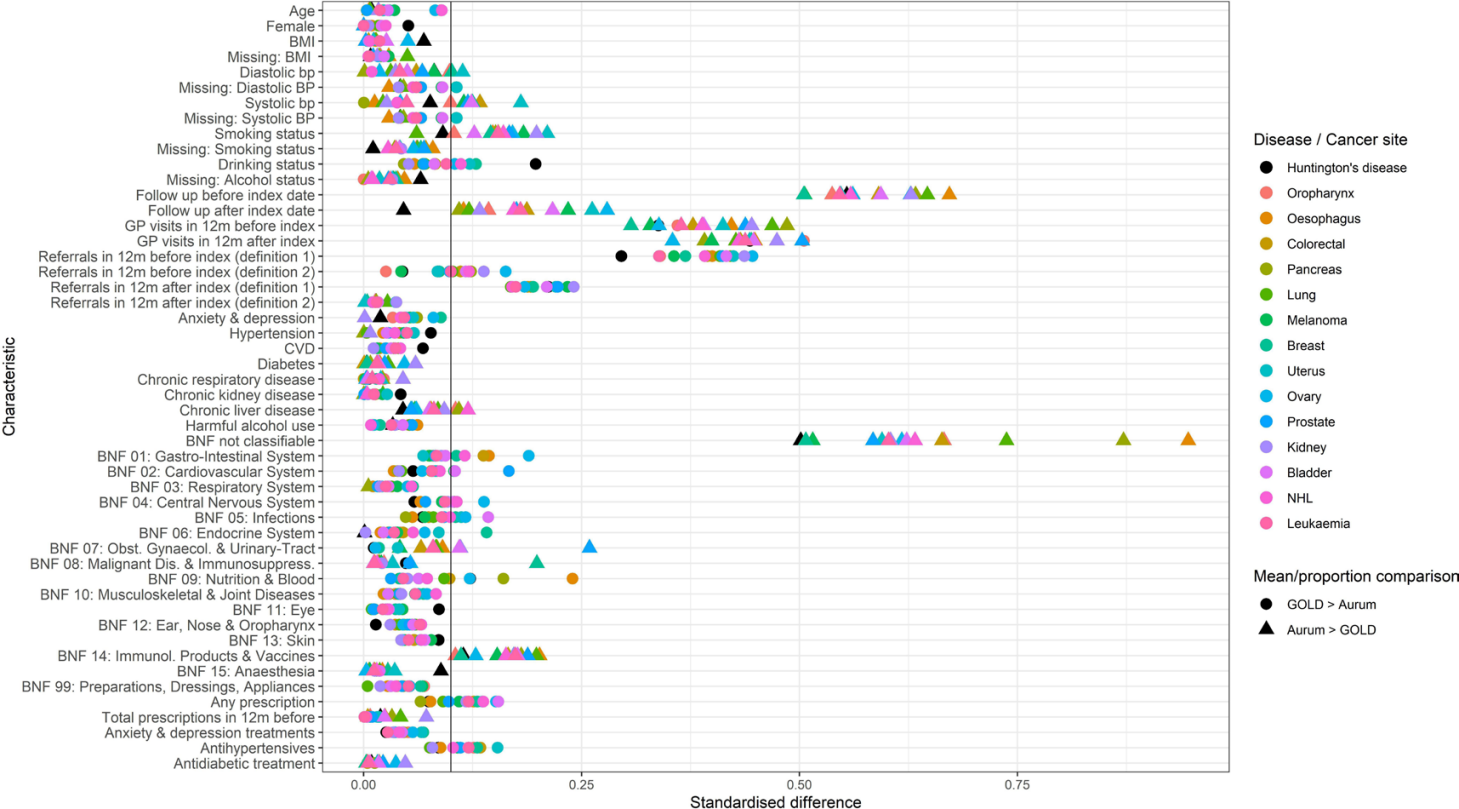
Standardised differences for baseline characteristics of incident Huntington’s disease (HD) and cancer patients in CPRD GOLD versus CPRD Aurum. Each row is a patient characteristic. Each point is a comparison between GOLD and Aurum for that characteristic in patients with an incident outcome. Symbol colour indicates outcome (ie, condition/cancer site). Symbol shape indicates direction of difference: circle symbol: mean/proportion highest in CPRD GOLD; triangle symbol: mean/proportion highest in CPRD Aurum. BMI, body mass index; BNF, British National Formulary; BP, blood pressure; CPRD, Clinical Practice Research Datalink; CVD, cardiovascular disease; GP, general practice.

Table 1 shows baseline characteristics of incident and prevalent HD cases in each database. Standardised mean differences are presented in figure 4. Average prior follow-up time for incident cases was longer in CPRD Aurum, reflecting the absence of a practice UTS date in that database. The medical history was similar in the two databases. Prescribing history in the 12 months prior to index date was also similar in terms of total prescriptions per patients, and the proportion of patients receiving at least one prescription. In CPRD Aurum, 7.3% of all prescriptions issued during the baseline period were assigned to the missing category (BNF chapter 00)—indicating either a missing, invalid or unmappable dm+d code in the product dictionary. The corresponding figure CPRD GOLD was just 1.3% (data not shown). This likely explains why the proportion of patients receiving a prescription from any given BNF chapter tended to be slightly higher in CPRD GOLD versus CPRD Aurum. This difference was particularly marked for BNF chapter 9 (nutrition and blood), and it was noted that the CPRD Aurum drug dictionary contained many nutritional supplements with no valid dm+d code, and therefore, assigned to chapter 00 (unclassified), whereas in the CPRD GOLD dictionary, most nutritional supplements products had a valid dm+d code and were correctly assigned to BNF chapter 9. The exception to this pattern was for BNF chapter 14 (vaccines and related immunological products) where the proportion of patients receiving a prescription was highest in CPRD Aurum.

A large proportion of patients with HD (>40%) had a prior history of or recent treatment for anxiety and depression, and around 60% of incident cases and 75% of prevalent cases had received a prescription for drugs affecting the central nervous system. Previous prescriptions for tetrabenazine—one of very few treatments specifically indicated for HD—were recorded for 15% of prevalent cases but also for a small proportion of incident cases in both CPRD GOLD (6.6%) and CPRD Aurum (5.1%). This suggests there may occasionally be a significant lag between establishing an HD diagnosis and recording it using a specific code.

### Characteristics of patients with cancer

Characteristics of the 14 site-specific incident patients with cancer in CPRD GOLD and CPRD Aurum are presented in online supplemental tables S1–S14, with comparisons between the two databases summarised as standardised differences in figure 4. Grouping characteristics by type, and considering the median standardised differences, patients in the two databases were most comparable in terms of medical history (median standardised difference 0.03, IQR 0.01–0.03); demographics and health-related characteristics (median standardised difference 0.05 (IQR 0.01–0.09)); and treatment history (median standardised difference 0.06 (IQR 0.03–0.10)) (see online supplemental figure S2). As with the incident HD cohort, patients in CPRD Aurum cohorts were more likely to receive prescriptions for products in BNF chapter 14 (immunological products and vaccines), and particularly for BNF chapter 00 (unmapped products). Larger differences between databases were seen for healthcare contacts (median standardised difference 0.27 (IQR 0.10–0.41)), and database follow-up (median standardised difference 0.39 (IQR 0.19–0.56)) CPRD Aurum cohorts, had longer average follow-up before and after index date, likely reflecting the lack of UTS date in CPRD Aurum and the loss of English practices from CPRD GOLD during the latter half of the study period. CPRD Aurum patients had fewer recorded GP consultations before and after the index date. Referrals before and after index date were systematically higher in CPRD GOLD when only specific referral records were considered, but the two databases gave much more similar results when clinical event records with a code for referral were also included.

## Discussion

### Main findings

We explored trends in incidence and prevalence of HD, a rare condition, and 14 common cancers in the CPRD GOLD and CPRD Aurum primary care databases, and undertook detailed characterisation of patients with these conditions. Data were broadly comparable in the two databases. Recorded HD incidence and prevalence was slightly higher in CPRD GOLD but trends were very similar in both databases and highly consistent with previous estimates.^37^,^39^ Incidence of common cancers showed somewhat different trends in the two databases between 1990–2000, but was remarkably similar thereafter and was consistent with national reference rates, particularly when primary care and linked secondary care and mortality data were combined. Across all conditions studied, results from the two databases were most similar in terms of patients’ medical history and recent prescribing, but some differences were seen including numbers of GP visits (higher in CPRD Aurum), referrals (higher in CPRD GOLD) and follow-up (longer in CPRD Aurum).

### Research in context

We consider below possible mechanisms and sources of variability which may underlie differences seen between CPRD GOLD and CPRD Aurum.

First, there may be real differences in the source populations. Both CPRD GOLD and CPRD Aurum contain data collected from general population in the same primary care setting within the UK NHS,^5 13^ but differ significantly in their regional coverage. Only CPRD GOLD includes data from practices in Wales and Scotland,^5 13^ and regional coverage within England is also quite different, reflecting among other things the distribution of practices using the respective GP software.^40^ Regional coverage has changed over time as well, particularly in CPRD GOLD where the number of practices from England dropped substantially between 2012 and 2017, which likely explains the slightly shorter average follow-up time after index date in that database. There are minor differences in age and sex structure between the databases, but adjusting for these had little impact on patterns of incidence and prevalence.

A second potential source of variability is the GP software user interface and how this supports recording of accurately coded clinical information and reduces the need for users to record clinical information as free text (which is not included in CPRD databases). One UK study compared how GPs used 4 different software systems over a series of 163 real-life patient consultations,^41^ and found that GPs using EMIS systems recorded significantly fewer clinical codes per consultation (1.5) than GPs using Vision or iSoft systems (2.9). Other studies have also found differences in completeness and quality of data recording in databases derived from different GP software,^42 43^ including one study of cancer recording in primary care in the Netherlands.^44^ The lower incidence and prevalence of HD in CPRD Aurum could indicate that GPs using EMIS software are less likely to code this diagnosis and more likely to use free text.

The different clinical and drug dictionaries and coding systems in CPRD GOLD and CPRD Aurum provide a third challenge when defining comparable patient groups and their clinical characteristics. For HD, with only two highly specific codes in CPRD GOLD and four in CPRD Aurum, case definition was relatively straightforward. Deriving comparable definitions for other conditions, and prescribing history was more challenging. A combination of approaches was required to map, for example, 779 codes for the 14 cancer sites previously validated for CPRD GOLD to 2279 codes in CPRD Aurum. The very similar trends in incidence and prevalence from 2000 onwards suggest this approach was largely successful, though the slightly higher incidence of ovarian and kidney cancer in CPRD Aurum remains unexplained. Although both databases include dm+d codes in their prescribing formularies, this does not provide an adequate cross-map between the two: between one-third and one-half of dictionary entries have no match in the other dictionary. Therefore, we mapped dm+d to BNF chapter to group all prescriptions. Although the completeness of dm+d coding was somewhat lower in CPRD Aurum, the results suggest prescribing patterns are very similar in the two databases.

As well as differences in the user interface, Vision and EMIS Web GP software differs in the underlying data models used to represent and store data. As a result, there are differences in the data structures for CPRD GOLD and CPRD Aurum,^5 13^ posing a fourth challenge when defining comparable clinical characteristics in each database. For example, information about vaccinations and immunisation is represented differently in CPRD GOLD and CPRD Aurum, and the largest relative differences in prescribing frequency were seen for vaccine and related immunological products. Information about referrals is also recorded differently in the two databases, and consequently a simple approach of counting referral records leads to large apparent differences in estimated referral counts, but a more complex algorithm generates more comparable referral counts. We also found systematic differences in the number of GP visits in the two databases, with patients in GOLD appearing to consult more frequently than those in Aurum. The most plausible explanation is that these arise from differences in the way consultations are recorded in the two databases. In GOLD, all entries to a patient record generate a ‘consultation’ event whose type indicates whether it was a face-to-face encounter or telephone encounter or an administrative update for example.^5^ In CPRD Aurum, encounter type is harder to deduce because the information is sometimes missing or redacted, and because patient records can be updated without creating an associated consultation record.

Differences in data curation methods—such as availability of a practice-level UTS data quality filter in CPRD GOLD but not CPRD Aurum represent a fifth potential source of variability between databases. UTS acts as a filter to remove lower quality data from practices likely to be under-recording, and the effect is to increase incidence rates particularly prior to 2000. The impact of similar data curation measures was reported previously for mortality and incidence of cancer and myocardial infarction in another UK primary care database, THIN, which is also based on data from Vision software.^45 46^ However, we suspect data quality issues in the early years of computerisation were likely to be widespread and not limited to practices using Vision software. We note also that cancer incidence trends between 1990 and 2000 for the whole CPRD Aurum database (figure 2) more closely resemble the trends in overlapping CPRD Aurum practices (figure 3) where UTS was not applied. Further research is needed to understand the impact of applying a filter similar to UTS date in CPRD Aurum. Researchers should be aware of the potential for differential data quality in the two databases, particularly between 1900 and 2000, and consider whether it is appropriate to combine data for this period.

Because CPRD Aurum is relatively new there are few published comparisons with CPRD GOLD. One study found that baseline characteristics of patients with chronic obstructive pulmonary disease (COPD) in 2017 were broadly comparable in the two databases in terms of patient demographics, BMI, smoking status, history of selected medical history, COPD severity and treatment patterns.^17^ A second study found that antibiotic prescribing rates for 25 common antibiotics were similar in each database during 2017, particularly when analyses were restricted to England only although the authors recommended further research to understand data quality and completeness around dosage regimens and treatment duration.^15^ Another study produced conflicting estimates and temporal trends in the incidence and prevalence of low back pain and osteoarthritis between 2005 and 2019 in CPRD Aurum and CPRD GOLD, and called for further research to understand the impact of analytical decisions and data quality on database heterogeneity.^16^ Other studies have combined data from CPRD GOLD and CPRD Aurum without presenting direct comparisons between the databases, making it difficult to gauge the potential impact of heteregeneity. These include studies of incidence and prevalence study of rare conditions including juvenile idiopathic arthritis between 2000 and 2018,^47^ and neuromuscular conditions between 2000 and 2019^48^; and a multicountry study examining the impact of a regulatory intervention to reduce inappropriate prescribing of mirabegron (a treatment for overactive bladder) to new users with severe or uncontrolled hypertension.^49^

Other studies have used and compared data from CPRD GOLD and the QResearch database, which is also derived from EMIS primary care systems, and found baseline characteristics in patients with other conditions to be generally similar.^50^,^54^

### Strengths and limitations

To our knowledge, this is the first published comparison of CPRD GOLD and CPRD Aurum to include both long-term incidence trends and detailed patient characteristics for specific conditions, and the first to include comparisons from a subset of overlapping practices in each database.

Migration of EHR data from one clinical system to another in overlapping practices must occur with high fidelity, which is essential to ensure that migrated records continue to support safe and effective clinical management of patients. Under this assumption, overlapping practices provide a setting to assess consistency of codelists and feature extraction algorithms across the two databases. The very similar incidence and prevalence of HD, and incidence of specific cancers in overlapping practices, therefore, provide reassurance that our case definitions were comparable. Because HD is a rare disease we were able to perform a simple probabilistic linkage of HD cases from overlapping practices and further demonstrate a high degree of concordance in case identification and classification in each database.

A limitation of our study was the lack of a gold-standard method for validating HD or cancer outcomes, and where differences were found between the databases we cannot say whether one is more correct. Even where results were very similar the case definitions may misclassify patients in both databases—for example, evidence of tetrabenazine prescribing in a small number of patients prior to the index date indicates a possible limitation in our definition of incident HD cases. We showed that for most cancer sites, incidence estimated from primary care data alone was consistent with national rates for England. However, both databases underestimated incidence of colorectal, lung, pancreas, uterine and kidney cancers compared with reference rates. This is consistent with previous reports of low sensitivity of primary care data for identifying cases in these sites.^33 55^ Combining primary care and linked data overestimated incidence of oesophageal, ovarian and bladder cancers, non-Hodgkin lymphoma (NHL) and leukaemia relative to national reference rates. This is consistent with the primary and secondary care data sources each having a relatively high sensitivity but relatively low positive predictive value.^33^ Our findings are also specific for HD and the selected cancer outcomes and are not necessarily generalisable to other conditions or other databases.

## Conclusions

Our study focused on identifying potential sources of heterogeneity in two UK primary care EHR databases, to guide decisions on whether to combine the data for further analysis and how to properly account for heterogeneity if present.^2 3^ The latter involves selection of appropriate statistical models, presenting results of each database separately in addition to pooled results, and design of relevant sensitivity and subgroup analyses. Our comparisons of incidence trends and patient characteristics suggest that we were able to identify similar groups of patients in each database, making it feasible to pool data to increase statistical power for a study of the association between cancer risk in patients with HD. Further research is needed to understand observed differences in measures of healthcare contacts. Differences in HD and cancer incidence trends were seen particularly between 1990 and 2000. This may reflect in part the impact of the UTS data quality filter which is applied to CPRD GOLD but not CPRD Aurum, but further research is needed to understand data quality in CPRD Aurum during this period. Greater caution is particularly warranted when considering whether to combine data prior to 2000. Similar investigations should be undertaken as a preliminary step in any study aiming to combine data from these databases.

## Supplementary material

10.1136/bmjopen-2022-070258 online supplemental file 1

## Data Availability

Data may be obtained from a third party and are not publicly available.
