# Correction: Pooling of primary care electronic health record (EHR) data on Huntington’s disease (HD) and cancer: establishing comparability of two large UK databases

**DOI:** 10.1136/bmjopen-2022-070258corr1

**Published:** 2025-06-16

 Dedman D, Williams R, Bhaskaran K, *et al*. Pooling of primary care electronic health record (EHR) data on Huntington’s disease (HD) and cancer: establishing comparability of two large UK databases. *BMJ Open* 2024;14:e070258. doi: 10.1136/bmjopen-2022-070258

This article was previously published with an error.

The word ‘million’ was incorrectly changed to ‘months’ in the first sentence of the final paragraph under the Data Sources section under Methods. The corrected text should read:

Analyses were conducted using the November 2020 build of the CPRD GOLD and CPRD Aurum databases, containing around 20 million and 40 million patient records, respectively, and the most contemporaneous linked data available at the time.

