# Supplementary material for: Pooling of primary care electronic health record (EHR) data on Huntington’s disease (HD) and cancer: establishing comparability of two large UK databases
Source: BMJ Open. 2024 Feb 14;14(2):e070258. doi: 10.1136/bmjopen-2022-070258 (PMC10868307; doi:10.1136/bmjopen-2022-070258)
Supplement: online supplemental file 1 [file bmjopen-2022-070258supp001.pdf]

# Feasibility of pooling patient data from multiple electronic health record (EHR) databases: Case studies in UK primary care

**Data supplement**

Figure S1: Huntington's disease incidence and prevalence by age group and region, 1990 – 2019: CPRD GOLD & CPRD Aurum

Table S1 – S14: characteristics of patients with incident diagnosis of 14 common cancers.

Figure S2: Boxplot summary of standardised differences in incident HD and cancer patients in CPRD GOLD and CPRD Aurum, for different types* of patient characteristic.

Appendix 1: Codelist Details

Figure S1: Huntington's disease incidence and prevalence by age group and region, 1990 – 2019: CPRD GOLD & CPRD Aurum

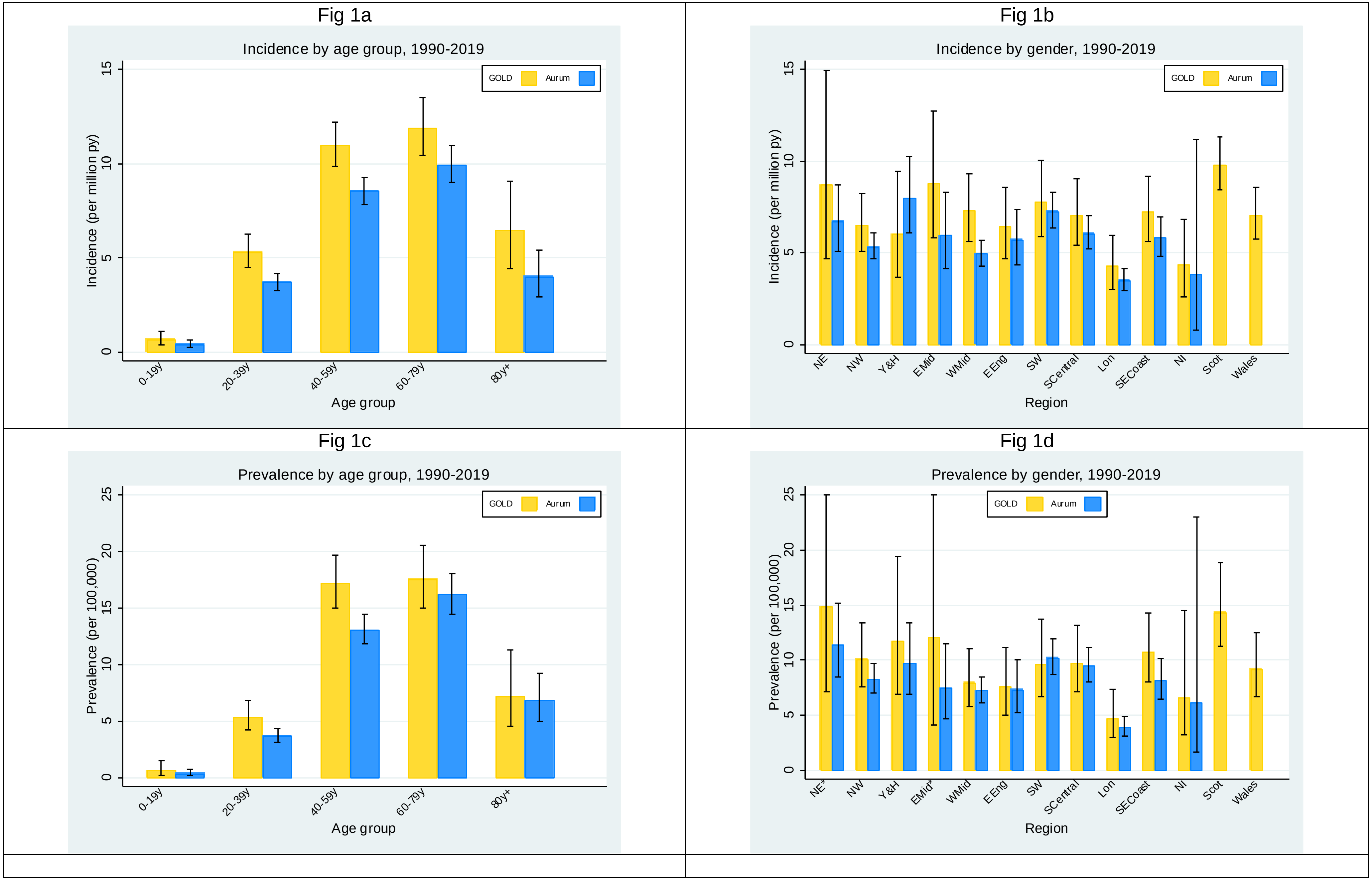

## Table S1: Malignant neoplasm of lip, oral cavity and pharynx (ICD-10 C00-C14): characteristics of patients with incident diagnosis in CPRD GOLD and CPRD Aurum

| | Incident cases | | |
|---|---|---|---|
| | CPRD Aurum | CPRD GOLD | *std.diff*[b] |
| **Total patients [n]** | 18875 | 10035 | |
| Age at index date [mean (sd)] | 63.4 (13.5) | 64.6 (13.4) | *0.09* |
| Female [n (%)] | 6536 (34.6) | 3560 (35.5) | *0.02* |
| BMI[a] [mean (sd)] | 25.5 (5.4) | 25.4 (4.5) | *0.01* |
| Missing BMI [n (%)] | 13174 (69.8) | 6915 (68.9) | *0.02* |
| Diastolic blood pressure[a] mmHg [mean (sd)] | 80.1 (10.7) | 79.0 (10.5) | *0.1* |
| Missing diastolic bp [n (%)] | 15589 (82.6) | 8445 (84.2) | *0.04* |
| Systolic blood pressure[a] mmHg [mean (sd)] | 135.0 (20.1) | 133.0 (19.0) | *0.1* |
| Missing systolic bp [n (%)] | 15589 (82.6) | 8445 (84.2) | *0.04* |
| Smoking[a]: current/ex [n (%)] | 14104 (77.7) | 7149 (73.3) | *0.1* |
| Missing smoking status [n (%)] | 726 (3.8) | 277 (2.8) | *0.06* |
| Alcohol status[a]: current/ex [n (%)] | 14894 (87.8) | 8148 (90.4) | *0.08* |
| Missing alcohol status [n (%)] | 1912 (10.1) | 1017 (10.1) | *<0.01* |
| Years of follow up prior to index date [mean (sd)] | 12.3 (8.1) | 8.5 (5.9) | *0.54* |
| Years of follow up after index date [mean (sd)] | 5.1 (5.5) | 4.4 (4.5) | *0.14* |
| **Healthcare contacts** | | | |
| GP visits in 12m before index date [n (sd)] | 9.5 (10.0) | 13.4 (11.6) | *0.36* |
| GP visits in 12m after index date [n (sd)] | 13.9 (13.9) | 22.0 (17.7) | *0.51* |
| Referrals (definition 1) in 12m before index date [n (sd)] | 0.7 (1.0) | 1.1 (1.3) | *0.34* |
| Referrals (definition 1) in 12m after index date [n (sd)] | 1.3 (1.6) | 1.3 (1.6) | *0.03* |
| Referrals (definition 2) in 12m before index date [n (sd)] | 0.4 (1.0) | 0.6 (1.3) | *0.19* |
| Referrals (definition 2) in 12m after index date [n (sd)] | 0.9 (1.6) | 0.9 (1.6) | *0.01* |
| **Medical history** | | | |
| Anxiety & depression [n (%)] | 5252 (27.8) | 2945 (29.3) | *0.03* |
| Hypertension [n (%)] | 6253 (33.1) | 3555 (35.4) | *0.05* |
| Cardiovascular disease [n (%)] | 3415 (18.1) | 1954 (19.5) | *0.04* |
| Diabetes [n (%)] | 1922 (10.2) | 1011 (10.1) | *<0.01* |
| Chronic respiratory disease [n (%)] | 3258 (17.3) | 1822 (18.2) | *0.02* |
| Chronic kidney disease [n (%)] | 1238 (6.6) | 692 (6.9) | *0.01* |
| Chronic liver disease [n (%)] | 423 (2.2) | 93 (0.9) | *0.11* |
| Alcohol: heavy or harmful use [n (%)] | 3221 (17.1) | 1913 (19.1) | *0.05* |
| **Treatment history: prescriptions in 12m prior to index date By BNF Chapter** | | | |
| 00: Not classifiable [n (%)] | 5291 (28.0) | 469 (4.7) | *0.67* |
| 01: Gastro-Intestinal System [n (%)] | 7067 (37.4) | 4148 (41.3) | *0.08* |
| 02: Cardiovascular System [n (%)] | 9088 (48.1) | 5255 (52.4) | *0.08* |
| 03: Respiratory System [n (%)] | 4293 (22.7) | 2399 (23.9) | *0.03* |
| 04: Central Nervous System [n (%)] | 10161 (53.8) | 5923 (59.0) | *0.1* |
| 05: Infections [n (%)] | 10090 (53.5) | 5813 (57.9) | *0.09* |
| 06: Endocrine System [n (%)] | 4420 (23.4) | 2531 (25.2) | *0.04* |
| 07: Obstetrics, Gynaecology and Urinary-Tract Disorders [n (%)] | 1798 (9.5) | 838 (8.4) | *0.04* |
| 08: Malignant Disease and Immunosuppression [n (%)] | 391 (2.1) | 176 (1.8) | *0.02* |
| 09: Nutrition and Blood [n (%)] | 4325 (22.9) | 2727 (27.2) | *0.1* |
| 10: Musculoskeletal and Joint Diseases [n (%)] | 5066 (26.8) | 2857 (28.5) | *0.04* |
| 11: Eye [n (%)] | 2037 (10.8) | 1227 (12.2) | *0.04* |
| 12: Ear, Nose and Oropharynx [n (%)] | 5016 (26.6) | 2867 (28.6) | *0.04* |
| 13: Skin [n (%)] | 4797 (25.4) | 2864 (28.5) | *0.07* |
| 14: Immunological Products and Vaccines [n (%)] | 2545 (13.5) | 1013 (10.1) | *0.11* |
| 15: Anaesthesia [n (%)] | 347 (1.8) | 205 (2.0) | *0.01* |
| 99: Other Preparations, Dressings, Appliances [n (%)] | 3385 (17.9) | 2075 (20.7) | *0.07* |
| Any prescription [n (%)] | 17005 (90.1) | 9414 (93.8) | *0.14* |
| Total prescriptions in 12m before index date [mean (sd)] | 41.8 (62.6) | 41.3 (53.2) | *0.01* |
| **Other treatments** | | | |
| Anxiety & depression treatments [n (%)] | 3939 (20.9) | 2308 (23.0) | *0.05* |
| Anti-hypertensives [n (%)] | 4862 (25.8) | 3157 (31.5) | *0.13* |
| Antidiabetic treatment [n (%)] | 1384 (7.3) | 728 (7.3) | *<0.01* |

[a]Measurement up to 3 years prior to index date.
[b]Standardised difference

## Table S2: Malignant neoplasm of oesophagus (ICD-10 C15): characteristics of patients with incident diagnosis in CPRD GOLD and CPRD Aurum

| | Incident cases | | |
|---|---|---|---|
| | CPRD Aurum | CPRD GOLD | *std.diff*[b] |
| **Total patients [n]** | 31175 | 15179 | |
| Age at index date [mean (sd)] | 71.0 (11.6) | 71.0 (11.5) | *0.01* |
| Female [n (%)] | 9968 (32.0) | 4920 (32.4) | *0.01* |
| BMI[a] [mean (sd)] | 26.1 (5.2) | 26.2 (4.6) | *0.01* |
| Missing BMI [n (%)] | 25217 (80.9) | 12105 (79.7) | *0.03* |
| Diastolic blood pressure[a] mmHg [mean (sd)] | 79.9 (10.3) | 79.5 (9.6) | *0.04* |
| Missing diastolic bp [n (%)] | 28167 (90.4) | 13581 (89.5) | *0.03* |
| Systolic blood pressure[a] mmHg [mean (sd)] | 136.9 (19.5) | 136.6 (18.9) | *0.01* |
| Missing systolic bp [n (%)] | 28167 (90.4) | 13581 (89.5) | *0.03* |
| Smoking[a]: current/ex [n (%)] | 22791 (78.1) | 10384 (71.7) | *0.15* |
| Missing smoking status [n (%)] | 2000 (6.4) | 699 (4.6) | *0.08* |
| Alcohol status[a]: current/ex [n (%)] | 23144 (86.4) | 11734 (88.3) | *0.06* |
| Missing alcohol status [n (%)] | 4384 (14.1) | 1894 (12.5) | *0.05* |
| Years of follow up prior to index date [mean (sd)] | 13.6 (8.1) | 8.8 (6.0) | *0.67* |
| Years of follow up after index date [mean (sd)] | 1.8 (3.3) | 1.5 (2.5) | *0.11* |
| **Healthcare contacts** | | | |
| GP visits in 12m before index date [n (sd)] | 21.7 (17.2) | 15.3 (12.5) | *0.42* |
| GP visits in 12m after index date [n (sd)] | 31.9 (23.1) | 22.6 (18.4) | *0.45* |
| Referrals (definition 1) in 12m before index date [n (sd)] | 0.8 (1.1) | 1.3 (1.5) | *0.4* |
| Referrals (definition 1) in 12m after index date [n (sd)] | 1.5 (1.6) | 1.6 (1.8) | *0.09* |
| Referrals (definition 2) in 12m before index date [n (sd)] | 0.4 (1.0) | 0.6 (1.4) | *0.19* |
| Referrals (definition 2) in 12m after index date [n (sd)] | 0.9 (1.7) | 0.9 (1.7) | *0.01* |
| **Medical history** | | | |
| Anxiety & depression [n (%)] | 7420 (23.8) | 3919 (25.8) | *0.05* |
| Hypertension [n (%)] | 13120 (42.1) | 6557 (43.2) | *0.02* |
| Cardiovascular disease [n (%)] | 7921 (25.4) | 3979 (26.2) | *0.02* |
| Diabetes [n (%)] | 4468 (14.3) | 2077 (13.7) | *0.02* |
| Chronic respiratory disease [n (%)] | 6205 (19.9) | 2961 (19.5) | *0.01* |
| Chronic kidney disease [n (%)] | 3499 (11.2) | 1703 (11.2) | *<0.01* |
| Chronic liver disease [n (%)] | 277 (0.9) | 45 (0.3) | *0.08* |
| Alcohol: heavy or harmful use [n (%)] | 2527 (8.1) | 1499 (9.9) | *0.06* |
| **Treatment history: prescriptions in 12m prior to index date By BNF Chapter** | | | |
| 00: Not classifiable [n (%)] | 13085 (42.0) | 846 (5.6) | *0.95* |
| 01: Gastro-Intestinal System [n (%)] | 22864 (73.3) | 12058 (79.4) | *0.14* |
| 02: Cardiovascular System [n (%)] | 19256 (61.8) | 9630 (63.4) | *0.03* |
| 03: Respiratory System [n (%)] | 7961 (25.5) | 3949 (26.0) | *0.01* |
| 04: Central Nervous System [n (%)] | 18445 (59.2) | 9464 (62.3) | *0.07* |
| 05: Infections [n (%)] | 13331 (42.8) | 6913 (45.5) | *0.06* |
| 06: Endocrine System [n (%)] | 8929 (28.6) | 4481 (29.5) | *0.02* |
| 07: Obstetrics, Gynaecology and Urinary-Tract Disorders [n (%)] | 3407 (10.9) | 1255 (8.3) | *0.09* |
| 08: Malignant Disease and Immunosuppression [n (%)] | 752 (2.4) | 337 (2.2) | *0.01* |
| 09: Nutrition and Blood [n (%)] | 9267 (29.7) | 6240 (41.1) | *0.24* |
| 10: Musculoskeletal and Joint Diseases [n (%)] | 8135 (26.1) | 4114 (27.1) | *0.02* |
| 11: Eye [n (%)] | 3956 (12.7) | 2057 (13.6) | *0.03* |
| 12: Ear, Nose and Oropharynx [n (%)] | 4140 (13.3) | 2207 (14.5) | *0.04* |
| 13: Skin [n (%)] | 8280 (26.6) | 4422 (29.1) | *0.06* |
| 14: Immunological Products and Vaccines [n (%)] | 6011 (19.3) | 1820 (12.0) | *0.2* |
| 15: Anaesthesia [n (%)] | 618 (2.0) | 257 (1.7) | *0.02* |
| 99: Other Preparations, Dressings, Appliances [n (%)] | 6077 (19.5) | 3127 (20.6) | *0.03* |
| Any prescription [n (%)] | 29916 (96.0) | 14776 (97.3) | *0.08* |
| Total prescriptions in 12m before index date [mean (sd)] | 48.2 (59.7) | 46.8 (53.0) | *0.02* |
| **Other treatments** | | | |
| Anxiety & depression treatments [n (%)] | 5715 (18.3) | 2996 (19.7) | *0.04* |
| Anti-hypertensives [n (%)] | 10974 (35.2) | 5992 (39.5) | *0.09* |
| Antidiabetic treatment [n (%)] | 3200 (10.3) | 1539 (10.1) | *<0.01* |

[a]Measurement up to 3 years prior to index date.
[b]Standardised difference

## Table S3: Malignant neoplasm of colon and rectum (ICD-10 C18-C20): characteristics of patients with incident diagnosis in CPRD GOLD and CPRD Aurum

| | Incident cases | | |
|---|---|---|---|
| | CPRD Aurum | CPRD GOLD | *std.diff*[b] |
| **Total patients [n]** | 117701 | 54113 | |
| Age at index date [mean (sd)] | 70.6 (12.3) | 70.9 (12.0) | *0.02* |
| Female [n (%)] | 52713 (44.8) | 24323 (44.9) | *<0.01* |
| BMI[a] [mean (sd)] | 26.4 (4.7) | 26.4 (4.5) | *0.01* |
| Missing BMI [n (%)] | 85674 (72.8) | 39874 (73.7) | *0.02* |
| Diastolic blood pressure[a] mmHg [mean (sd)] | 80.8 (10.5) | 80.2 (9.8) | *0.06* |
| Missing diastolic bp [n (%)] | 101402 (86.2) | 47796 (88.3) | *0.07* |
| Systolic blood pressure[a] mmHg [mean (sd)] | 139.1 (20.9) | 136.4 (19.1) | *0.13* |
| Missing systolic bp [n (%)] | 101402 (86.2) | 47796 (88.3) | *0.07* |
| Smoking[a]: current/ex [n (%)] | 76391 (68.3) | 31816 (61.0) | *0.15* |
| Missing smoking status [n (%)] | 5897 (5.0) | 1930 (3.6) | *0.07* |
| Alcohol status[a]: current/ex [n (%)] | 87540 (84.7) | 42190 (87.6) | *0.08* |
| Missing alcohol status [n (%)] | 14358 (12.2) | 5924 (10.9) | *0.04* |
| Years of follow up prior to index date [mean (sd)] | 13.0 (8.2) | 8.7 (6.0) | *0.59* |
| Years of follow up after index date [mean (sd)] | 4.9 (5.5) | 3.9 (4.3) | *0.19* |
| **Healthcare contacts** | | | |
| GP visits in 12m before index date [n (sd)] | 20.0 (16.7) | 14.6 (11.9) | *0.38* |
| GP visits in 12m after index date [n (sd)] | 29.6 (22.1) | 21.1 (17.0) | *0.43* |
| Referrals (definition 1) in 12m before index date [n (sd)] | 0.7 (1.1) | 1.3 (1.5) | *0.39* |
| Referrals (definition 1) in 12m after index date [n (sd)] | 1.3 (1.6) | 1.5 (1.7) | *0.11* |
| Referrals (definition 2) in 12m before index date [n (sd)] | 0.4 (1.0) | 0.6 (1.4) | *0.19* |
| Referrals (definition 2) in 12m after index date [n (sd)] | 0.9 (1.6) | 0.9 (1.7) | *0.01* |
| **Medical history** | | | |
| Anxiety & depression [n (%)] | 26924 (22.9) | 13631 (25.2) | *0.05* |
| Hypertension [n (%)] | 48343 (41.1) | 23326 (43.1) | *0.04* |
| Cardiovascular disease [n (%)] | 25644 (21.8) | 12564 (23.2) | *0.03* |
| Diabetes [n (%)] | 16276 (13.8) | 7471 (13.8) | *<0.01* |
| Chronic respiratory disease [n (%)] | 19268 (16.4) | 8862 (16.4) | *<0.01* |
| Chronic kidney disease [n (%)] | 12264 (10.4) | 5865 (10.8) | *0.01* |
| Chronic liver disease [n (%)] | 657 (0.6) | 53 (0.1) | *0.08* |
| Alcohol: heavy or harmful use [n (%)] | 5405 (4.6) | 3160 (5.8) | *0.06* |
| **Treatment history: prescriptions in 12m prior to index date By BNF Chapter** | | | |
| 00: Not classifiable [n (%)] | 32895 (27.9) | 2535 (4.7) | *0.66* |
| 01: Gastro-Intestinal System [n (%)] | 65580 (55.7) | 33801 (62.5) | *0.14* |
| 02: Cardiovascular System [n (%)] | 67782 (57.6) | 33222 (61.4) | *0.08* |
| 03: Respiratory System [n (%)] | 24369 (20.7) | 11851 (21.9) | *0.03* |
| 04: Central Nervous System [n (%)] | 59136 (50.2) | 29966 (55.4) | *0.1* |
| 05: Infections [n (%)] | 46852 (39.8) | 23531 (43.5) | *0.07* |
| 06: Endocrine System [n (%)] | 31662 (26.9) | 15660 (28.9) | *0.05* |
| 07: Obstetrics, Gynaecology and Urinary-Tract Disorders [n (%)] | 12809 (10.9) | 4827 (8.9) | *0.07* |
| 08: Malignant Disease and Immunosuppression [n (%)] | 2944 (2.5) | 1216 (2.2) | *0.02* |
| 09: Nutrition and Blood [n (%)] | 35970 (30.6) | 19042 (35.2) | *0.1* |
| 10: Musculoskeletal and Joint Diseases [n (%)] | 28608 (24.3) | 13775 (25.5) | *0.03* |
| 11: Eye [n (%)] | 14154 (12.0) | 6996 (12.9) | *0.03* |
| 12: Ear, Nose and Oropharynx [n (%)] | 12592 (10.7) | 6833 (12.6) | *0.06* |
| 13: Skin [n (%)] | 30016 (25.5) | 15200 (28.1) | *0.06* |
| 14: Immunological Products and Vaccines [n (%)] | 21606 (18.4) | 6710 (12.4) | *0.17* |
| 15: Anaesthesia [n (%)] | 2208 (1.9) | 923 (1.7) | *0.01* |
| 99: Other Preparations, Dressings, Appliances [n (%)] | 23209 (19.7) | 11678 (21.6) | *0.05* |
| Any prescription [n (%)] | 108135 (91.9) | 51405 (95.0) | *0.13* |
| Total prescriptions in 12m before index date [mean (sd)] | 42.1 (55.4) | 41.8 (48.2) | *<0.01* |
| **Other treatments** | | | |
| Anxiety & depression treatments [n (%)] | 19278 (16.4) | 9808 (18.1) | *0.05* |
| Anti-hypertensives [n (%)] | 37143 (31.6) | 20526 (37.9) | *0.13* |
| Antidiabetic treatment [n (%)] | 11691 (9.9) | 5446 (10.1) | *<0.01* |

[a]Measurement up to 3 years prior to index date.
[b]Standardised difference

## Table S4: Malignant neoplasm of pancreas (ICD-10 C25): characteristics of patients with incident diagnosis in CPRD GOLD and CPRD Aurum

| | Incident cases | | |
|---|---|---|---|
| | CPRD Aurum | CPRD GOLD | *std.diff*[b] |
| **Total patients [n]** | 22872 | 10417 | |
| Age at index date [mean (sd)] | 71.7 (11.6) | 71.8 (11.4) | *<0.01* |
| Female [n (%)] | 11308 (49.4) | 5248 (50.4) | *0.02* |
| BMI[a] [mean (sd)] | 26.1 (4.7) | 26.1 (4.4) | *0.01* |
| Missing BMI [n (%)] | 18821 (82.3) | 8501 (81.6) | *0.02* |
| Diastolic blood pressure[a] mmHg [mean (sd)] | 79.7 (9.5) | 79.7 (9.6) | *<0.01* |
| Missing diastolic bp [n (%)] | 21176 (92.6) | 9515 (91.3) | *0.05* |
| Systolic blood pressure[a] mmHg [mean (sd)] | 135.9 (18.9) | 135.9 (19.1) | *<0.01* |
| Missing systolic bp [n (%)] | 21176 (92.6) | 9515 (91.3) | *0.05* |
| Smoking[a]: current/ex [n (%)] | 15647 (72.7) | 6499 (65.7) | *0.15* |
| Missing smoking status [n (%)] | 1345 (5.9) | 528 (5.1) | *0.04* |
| Alcohol status[a]: current/ex [n (%)] | 16823 (84.9) | 7824 (86.5) | *0.05* |
| Missing alcohol status [n (%)] | 3047 (13.3) | 1369 (13.1) | *0.01* |
| Years of follow up prior to index date [mean (sd)] | 13.7 (8.3) | 9.1 (6.1) | *0.63* |
| Years of follow up after index date [mean (sd)] | 1.0 (2.4) | 0.8 (1.6) | *0.11* |
| **Healthcare contacts** | | | |
| GP visits in 12m before index date [n (sd)] | 26.8 (18.9) | 18.8 (13.4) | *0.49* |
| GP visits in 12m after index date [n (sd)] | 27.2 (23.1) | 19.2 (17.7) | *0.39* |
| Referrals (definition 1) in 12m before index date [n (sd)] | 0.9 (1.3) | 1.6 (1.9) | *0.42* |
| Referrals (definition 1) in 12m after index date [n (sd)] | 1.7 (1.9) | 2.0 (2.2) | *0.12* |
| Referrals (definition 2) in 12m before index date [n (sd)] | 0.4 (0.9) | 0.6 (1.2) | *0.19* |
| Referrals (definition 2) in 12m after index date [n (sd)] | 0.8 (1.5) | 0.8 (1.5) | *0.01* |
| **Medical history** | | | |
| Anxiety & depression [n (%)] | 6126 (26.8) | 3078 (29.5) | *0.06* |
| Hypertension [n (%)] | 10137 (44.3) | 4616 (44.3) | *<0.01* |
| Cardiovascular disease [n (%)] | 5532 (24.2) | 2632 (25.3) | *0.03* |
| Diabetes [n (%)] | 5785 (25.3) | 2600 (25.0) | *0.01* |
| Chronic respiratory disease [n (%)] | 4076 (17.8) | 1771 (17.0) | *0.02* |
| Chronic kidney disease [n (%)] | 2724 (11.9) | 1240 (11.9) | *<0.01* |
| Chronic liver disease [n (%)] | 207 (0.9) | 13 (0.1) | *0.11* |
| Alcohol: heavy or harmful use [n (%)] | 1184 (5.2) | 648 (6.2) | *0.05* |
| **Treatment history: prescriptions in 12m prior to index date By BNF Chapter** | | | |
| 00: Not classifiable [n (%)] | 9010 (39.4) | 619 (5.9) | *0.87* |
| 01: Gastro-Intestinal System [n (%)] | 16368 (71.6) | 7832 (75.2) | *0.08* |
| 02: Cardiovascular System [n (%)] | 14831 (64.8) | 6949 (66.7) | *0.04* |
| 03: Respiratory System [n (%)] | 6414 (28.0) | 2896 (27.8) | *0.01* |
| 04: Central Nervous System [n (%)] | 16115 (70.5) | 7822 (75.1) | *0.1* |
| 05: Infections [n (%)] | 10960 (47.9) | 5244 (50.3) | *0.05* |
| 06: Endocrine System [n (%)] | 8984 (39.3) | 4235 (40.7) | *0.03* |
| 07: Obstetrics, Gynaecology and Urinary-Tract Disorders [n (%)] | 2857 (12.5) | 1026 (9.8) | *0.08* |
| 08: Malignant Disease and Immunosuppression [n (%)] | 593 (2.6) | 241 (2.3) | *0.02* |
| 09: Nutrition and Blood [n (%)] | 6405 (28.0) | 3693 (35.5) | *0.16* |
| 10: Musculoskeletal and Joint Diseases [n (%)] | 7177 (31.4) | 3412 (32.8) | *0.03* |
| 11: Eye [n (%)] | 3096 (13.5) | 1455 (14.0) | *0.01* |
| 12: Ear, Nose and Oropharynx [n (%)] | 2820 (12.3) | 1449 (13.9) | *0.05* |
| 13: Skin [n (%)] | 6985 (30.5) | 3392 (32.6) | *0.04* |
| 14: Immunological Products and Vaccines [n (%)] | 4625 (20.2) | 1405 (13.5) | *0.18* |
| 15: Anaesthesia [n (%)] | 658 (2.9) | 289 (2.8) | *0.01* |
| 99: Other Preparations, Dressings, Appliances [n (%)] | 6101 (26.7) | 2957 (28.4) | *0.04* |
| Any prescription [n (%)] | 22158 (96.9) | 10200 (97.9) | *0.07* |
| Total prescriptions in 12m before index date [mean (sd)] | 50.9 (62.1) | 49.1 (50.2) | *0.03* |
| **Other treatments** | | | |
| Anxiety & depression treatments [n (%)] | 5215 (22.8) | 2528 (24.3) | *0.03* |
| Anti-hypertensives [n (%)] | 8269 (36.2) | 4158 (39.9) | *0.08* |
| Antidiabetic treatment [n (%)] | 4579 (20.0) | 2139 (20.5) | *0.01* |

[a]Measurement up to 3 years prior to index date.
[b]Standardised difference

## Table S5: Malignant neoplasm of bronchus and lung (ICD-10 C34): characteristics of patients with incident diagnosis in CPRD GOLD and CPRD Aurum

| | Incident cases | | |
|---|---|---|---|
| | CPRD Aurum | CPRD GOLD | *std.diff*[b] |
| **Total patients [n]** | 116446 | 56227 | |
| Age at index date [mean (sd)] | 71.5 (10.6) | 71.4 (10.5) | *0.01* |
| Female [n (%)] | 50478 (43.3) | 24856 (44.2) | *0.02* |
| BMI[a] [mean (sd)] | 25.5 (4.7) | 25.5 (4.4) | *0.01* |
| Missing BMI [n (%)] | 94531 (81.2) | 44521 (79.2) | *0.05* |
| Diastolic blood pressure[a] mmHg [mean (sd)] | 79.7 (10.2) | 79.4 (9.8) | *0.03* |
| Missing diastolic bp [n (%)] | 107321 (92.2) | 51158 (91.0) | *0.04* |
| Systolic blood pressure[a] mmHg [mean (sd)] | 136.9 (19.8) | 136.4 (19.1) | *0.02* |
| Missing systolic bp [n (%)] | 107321 (92.2) | 51158 (91.0) | *0.04* |
| Smoking[a]: current/ex [n (%)] | 100587 (91.7) | 48334 (90.0) | *0.06* |
| Missing smoking status [n (%)] | 6809 (5.8) | 2520 (4.5) | *0.06* |
| Alcohol status[a]: current/ex [n (%)] | 84311 (84.7) | 42201 (87.2) | *0.07* |
| Missing alcohol status [n (%)] | 16961 (14.6) | 7831 (13.9) | *0.02* |
| Years of follow up prior to index date [mean (sd)] | 13.4 (8.2) | 8.8 (6.0) | *0.65* |
| Years of follow up after index date [mean (sd)] | 1.5 (2.8) | 1.2 (2.1) | *0.12* |
| **Healthcare contacts** | | | |
| GP visits in 12m before index date [n (sd)] | 24.9 (18.1) | 17.5 (13.1) | *0.47* |
| GP visits in 12m after index date [n (sd)] | 27.4 (21.6) | 19.1 (17.0) | *0.43* |
| Referrals (definition 1) in 12m before index date [n (sd)] | 0.8 (1.2) | 1.4 (1.7) | *0.41* |
| Referrals (definition 1) in 12m after index date [n (sd)] | 1.5 (1.8) | 1.7 (1.9) | *0.1* |
| Referrals (definition 2) in 12m before index date [n (sd)] | 0.4 (1.0) | 0.6 (1.2) | *0.17* |
| Referrals (definition 2) in 12m after index date [n (sd)] | 0.9 (1.6) | 0.8 (1.5) | *0.03* |
| **Medical history** | | | |
| Anxiety & depression [n (%)] | 34437 (29.6) | 17866 (31.8) | *0.05* |
| Hypertension [n (%)] | 47448 (40.7) | 23014 (40.9) | *<0.01* |
| Cardiovascular disease [n (%)] | 34123 (29.3) | 17513 (31.1) | *0.04* |
| Diabetes [n (%)] | 16114 (13.8) | 7243 (12.9) | *0.03* |
| Chronic respiratory disease [n (%)] | 40520 (34.8) | 19463 (34.6) | *<0.01* |
| Chronic kidney disease [n (%)] | 14237 (12.2) | 6474 (11.5) | *0.02* |
| Chronic liver disease [n (%)] | 885 (0.8) | 100 (0.2) | *0.09* |
| Alcohol: heavy or harmful use [n (%)] | 8798 (7.6) | 5074 (9.0) | *0.05* |
| **Treatment history: prescriptions in 12m prior to index date By BNF Chapter** | | | |
| 00: Not classifiable [n (%)] | 40070 (34.4) | 3657 (6.5) | *0.74* |
| 01: Gastro-Intestinal System [n (%)] | 62112 (53.3) | 32096 (57.1) | *0.08* |
| 02: Cardiovascular System [n (%)] | 75503 (64.8) | 37623 (66.9) | *0.04* |
| 03: Respiratory System [n (%)] | 55618 (47.8) | 27759 (49.4) | *0.03* |
| 04: Central Nervous System [n (%)] | 80410 (69.1) | 41153 (73.2) | *0.09* |
| 05: Infections [n (%)] | 77181 (66.3) | 39367 (70.0) | *0.08* |
| 06: Endocrine System [n (%)] | 48021 (41.2) | 24213 (43.1) | *0.04* |
| 07: Obstetrics, Gynaecology and Urinary-Tract Disorders [n (%)] | 12602 (10.8) | 4716 (8.4) | *0.08* |
| 08: Malignant Disease and Immunosuppression [n (%)] | 3213 (2.8) | 1387 (2.5) | *0.02* |
| 09: Nutrition and Blood [n (%)] | 30870 (26.5) | 17251 (30.7) | *0.09* |
| 10: Musculoskeletal and Joint Diseases [n (%)] | 40348 (34.6) | 20549 (36.5) | *0.04* |
| 11: Eye [n (%)] | 15420 (13.2) | 7626 (13.6) | *0.01* |
| 12: Ear, Nose and Oropharynx [n (%)] | 18096 (15.5) | 9587 (17.1) | *0.04* |
| 13: Skin [n (%)] | 32430 (27.8) | 16868 (30.0) | *0.05* |
| 14: Immunological Products and Vaccines [n (%)] | 22590 (19.4) | 6863 (12.2) | *0.2* |
| 15: Anaesthesia [n (%)] | 2988 (2.6) | 1283 (2.3) | *0.02* |
| 99: Other Preparations, Dressings, Appliances [n (%)] | 30717 (26.4) | 14949 (26.6) | *<0.01* |
| Any prescription [n (%)] | 112453 (96.6) | 55129 (98.0) | *0.09* |
| Total prescriptions in 12m before index date [mean (sd)] | 57.0 (69.6) | 54.3 (58.4) | *0.04* |
| **Other treatments** | | | |
| Anxiety & depression treatments [n (%)] | 30581 (26.3) | 15886 (28.3) | *0.04* |
| Anti-hypertensives [n (%)] | 39515 (33.9) | 21127 (37.6) | *0.08* |
| Antidiabetic treatment [n (%)] | 11647 (10.0) | 5325 (9.5) | *0.02* |

[a]Measurement up to 3 years prior to index date.
[b]Standardised difference

## Table S6: Malignant melanoma of skin (ICD-10 C43): characteristics of patients with incident diagnosis in CPRD GOLD and CPRD Aurum

| | Incident cases | | |
|---|---|---|---|
| | CPRD Aurum | CPRD GOLD | *std.diff*[b] |
| **Total patients [n]** | 54775 | 23400 | |
| Age at index date [mean (sd)] | 61.0 (16.9) | 61.6 (16.6) | *0.04* |
| Female [n (%)] | 29585 (54.0) | 12676 (54.2) | *<0.01* |
| BMI[a] [mean (sd)] | 26.1 (4.6) | 26.0 (4.4) | *0.01* |
| Missing BMI [n (%)] | 34948 (63.8) | 15254 (65.2) | *0.03* |
| Diastolic blood pressure[a] mmHg [mean (sd)] | 78.9 (10.7) | 78.1 (10.2) | *0.08* |
| Missing diastolic bp [n (%)] | 44266 (80.8) | 19704 (84.2) | *0.09* |
| Systolic blood pressure[a] mmHg [mean (sd)] | 131.8 (19.6) | 129.7 (17.8) | *0.11* |
| Missing systolic bp [n (%)] | 44266 (80.8) | 19704 (84.2) | *0.09* |
| Smoking[a]: current/ex [n (%)] | 32602 (61.1) | 11981 (52.0) | *0.18* |
| Missing smoking status [n (%)] | 1376 (2.5) | 360 (1.5) | *0.07* |
| Alcohol status[a]: current/ex [n (%)] | 43476 (87.8) | 19235 (90.0) | *0.07* |
| Missing alcohol status [n (%)] | 5268 (9.6) | 2018 (8.6) | *0.03* |
| Years of follow up prior to index date [mean (sd)] | 12.6 (8.4) | 8.9 (6.1) | *0.51* |
| Years of follow up after index date [mean (sd)] | 6.9 (6.3) | 5.6 (4.9) | *0.23* |
| **Healthcare contacts** | | | |
| GP visits in 12m before index date [n (sd)] | 16.2 (15.2) | 11.9 (10.9) | *0.33* |
| GP visits in 12m after index date [n (sd)] | 22.8 (17.7) | 16.4 (13.7) | *0.4* |
| Referrals (definition 1) in 12m before index date [n (sd)] | 0.7 (1.0) | 1.1 (1.2) | *0.36* |
| Referrals (definition 1) in 12m after index date [n (sd)] | 1.3 (1.4) | 1.3 (1.4) | *0.04* |
| Referrals (definition 2) in 12m before index date [n (sd)] | 0.5 (1.0) | 0.7 (1.2) | *0.23* |
| Referrals (definition 2) in 12m after index date [n (sd)] | 0.9 (1.5) | 0.9 (1.4) | *0.01* |
| **Medical history** | | | |
| Anxiety & depression [n (%)] | 13355 (24.4) | 6246 (26.7) | *0.05* |
| Hypertension [n (%)] | 16792 (30.7) | 7679 (32.8) | *0.05* |
| Cardiovascular disease [n (%)] | 7466 (13.6) | 3318 (14.2) | *0.02* |
| Diabetes [n (%)] | 4447 (8.1) | 1877 (8.0) | *<0.01* |
| Chronic respiratory disease [n (%)] | 7354 (13.4) | 3156 (13.5) | *<0.01* |
| Chronic kidney disease [n (%)] | 4020 (7.3) | 1852 (7.9) | *0.02* |
| Chronic liver disease [n (%)] | 147 (0.3) | 9 (0.0) | *0.06* |
| Alcohol: heavy or harmful use [n (%)] | 1877 (3.4) | 883 (3.8) | *0.02* |
| **Treatment history: prescriptions in 12m prior to index date**<br>**By BNF Chapter** | | | |
| 00: Not classifiable [n (%)] | 10077 (18.4) | 698 (3.0) | *0.52* |
| 01: Gastro-Intestinal System [n (%)] | 15649 (28.6) | 7504 (32.1) | *0.08* |
| 02: Cardiovascular System [n (%)] | 23019 (42.0) | 11039 (47.2) | *0.1* |
| 03: Respiratory System [n (%)] | 8834 (16.1) | 4112 (17.6) | *0.04* |
| 04: Central Nervous System [n (%)] | 20274 (37.0) | 9686 (41.4) | *0.09* |
| 05: Infections [n (%)] | 20406 (37.3) | 9518 (40.7) | *0.07* |
| 06: Endocrine System [n (%)] | 12033 (22.0) | 5522 (23.6) | *0.04* |
| 07: Obstetrics, Gynaecology and Urinary-Tract Disorders [n (%)] | 7577 (13.8) | 2909 (12.4) | *0.04* |
| 08: Malignant Disease and Immunosuppression [n (%)] | 1248 (2.3) | 465 (2.0) | *0.02* |
| 09: Nutrition and Blood [n (%)] | 6483 (11.8) | 3087 (13.2) | *0.04* |
| 10: Musculoskeletal and Joint Diseases [n (%)] | 12297 (22.5) | 5841 (25.0) | *0.06* |
| 11: Eye [n (%)] | 5912 (10.8) | 2860 (12.2) | *0.04* |
| 12: Ear, Nose and Oropharynx [n (%)] | 5848 (10.7) | 2938 (12.6) | *0.06* |
| 13: Skin [n (%)] | 14570 (26.6) | 7043 (30.1) | *0.08* |
| 14: Immunological Products and Vaccines [n (%)] | 8326 (15.2) | 2368 (10.1) | *0.15* |
| 15: Anaesthesia [n (%)] | 2499 (4.6) | 1033 (4.4) | *0.01* |
| 99: Other Preparations, Dressings, Appliances [n (%)] | 10314 (18.8) | 5040 (21.5) | *0.07* |
| Any prescription [n (%)] | 46415 (84.7) | 20702 (88.5) | *0.11* |
| Total prescriptions in 12m before index date [mean (sd)] | 32.3 (49.2) | 32.6 (44.2) | *0.01* |
| **Other treatments** | | | |
| Anxiety & depression treatments [n (%)] | 8286 (15.1) | 3888 (16.6) | *0.04* |
| Anti-hypertensives [n (%)] | 13322 (24.3) | 6807 (29.1) | *0.11* |
| Antidiabetic treatment [n (%)] | 3250 (5.9) | 1373 (5.9) | *<0.01* |

[a]Measurement up to 3 years prior to index date.
[b]Standardised difference

## Table S7: Malignant neoplasm of breast (ICD-10 C50): characteristics of patients with incident diagnosis in CPRD GOLD and CPRD Aurum

| | Incident cases | | |
|---|---|---|---|
| | CPRD Aurum | CPRD GOLD | *std.diff*[b] |
| **Total patients [n]** | 187649 | 81709 | |
| Age at index date [mean (sd)] | 62.5 (14.3) | 63.0 (14.1) | *0.03* |
| Female [n (%)] | 187649 (100.0) | 81709 (100.0) | -- |
| BMI[a] [mean (sd)] | 26.2 (5.1) | 26.2 (5.1) | *<0.01* |
| Missing BMI [n (%)] | 118788 (63.3) | 52625 (64.4) | *0.02* |
| Diastolic blood pressure[a] mmHg [mean (sd)] | 79.4 (10.6) | 78.3 (10.1) | *0.1* |
| Missing diastolic bp [n (%)] | 150057 (80.0) | 68671 (84.0) | *0.11* |
| Systolic blood pressure[a] mmHg [mean (sd)] | 133.7 (21.2) | 131.1 (19.8) | *0.12* |
| Missing systolic bp [n (%)] | 150057 (80.0) | 68671 (84.0) | *0.11* |
| Smoking[a]: current/ex [n (%)] | 107316 (59.2) | 41499 (52.0) | *0.15* |
| Missing smoking status [n (%)] | 6417 (3.4) | 1900 (2.3) | *0.07* |
| Alcohol status[a]: current/ex [n (%)] | 133871 (79.6) | 62671 (84.5) | *0.13* |
| Missing alcohol status [n (%)] | 19399 (10.3) | 7550 (9.2) | *0.04* |
| Years of follow up prior to index date [mean (sd)] | 11.9 (8.0) | 8.3 (5.9) | *0.5* |
| Years of follow up after index date [mean (sd)] | 7.4 (6.4) | 5.9 (5.0) | *0.26* |
| **Healthcare contacts** | | | |
| GP visits in 12m before index date [n (sd)] | 14.5 (14.1) | 10.8 (10.0) | *0.31* |
| GP visits in 12m after index date [n (sd)] | 26.3 (18.6) | 18.9 (14.3) | *0.44* |
| Referrals (definition 1) in 12m before index date [n (sd)] | 0.6 (0.9) | 1.0 (1.2) | *0.37* |
| Referrals (definition 1) in 12m after index date [n (sd)] | 1.0 (1.4) | 1.2 (1.4) | *0.09* |
| Referrals (definition 2) in 12m before index date [n (sd)] | 0.4 (1.0) | 0.6 (1.3) | *0.19* |
| Referrals (definition 2) in 12m after index date [n (sd)] | 0.8 (1.6) | 0.8 (1.5) | *<0.01* |
| **Medical history** | | | |
| Anxiety & depression [n (%)] | 57604 (30.7) | 28483 (34.9) | *0.09* |
| Hypertension [n (%)] | 56071 (29.9) | 26607 (32.6) | *0.06* |
| Cardiovascular disease [n (%)] | 20223 (10.8) | 9625 (11.8) | *0.03* |
| Diabetes [n (%)] | 14452 (7.7) | 6197 (7.6) | *<0.01* |
| Chronic respiratory disease [n (%)] | 26812 (14.3) | 12106 (14.8) | *0.01* |
| Chronic kidney disease [n (%)] | 12205 (6.5) | 5556 (6.8) | *0.01* |
| Chronic liver disease [n (%)] | 627 (0.3) | 53 (0.1) | *0.06* |
| Alcohol: heavy or harmful use [n (%)] | 4812 (2.6) | 2349 (2.9) | *0.02* |
| **Treatment history: prescriptions in 12m prior to index date By BNF Chapter** | | | |
| 00: Not classifiable [n (%)] | 36034 (19.2) | 2929 (3.6) | *0.51* |
| 01: Gastro-Intestinal System [n (%)] | 55031 (29.3) | 28018 (34.3) | *0.11* |
| 02: Cardiovascular System [n (%)] | 76753 (40.9) | 36632 (44.8) | *0.08* |
| 03: Respiratory System [n (%)] | 35426 (18.9) | 17282 (21.2) | *0.06* |
| 04: Central Nervous System [n (%)] | 86138 (45.9) | 41727 (51.1) | *0.1* |
| 05: Infections [n (%)] | 70102 (37.4) | 34745 (42.5) | *0.11* |
| 06: Endocrine System [n (%)] | 53318 (28.4) | 28572 (35.0) | *0.14* |
| 07: Obstetrics, Gynaecology and Urinary-Tract Disorders [n (%)] | 19751 (10.5) | 9054 (11.1) | *0.02* |
| 08: Malignant Disease and Immunosuppression [n (%)] | 14917 (7.9) | 2762 (3.4) | *0.2* |
| 09: Nutrition and Blood [n (%)] | 26562 (14.2) | 12805 (15.7) | *0.04* |
| 10: Musculoskeletal and Joint Diseases [n (%)] | 46038 (24.5) | 22515 (27.6) | *0.07* |
| 11: Eye [n (%)] | 20206 (10.8) | 9755 (11.9) | *0.04* |
| 12: Ear, Nose and Oropharynx [n (%)] | 19390 (10.3) | 9880 (12.1) | *0.06* |
| 13: Skin [n (%)] | 43697 (23.3) | 21367 (26.2) | *0.07* |
| 14: Immunological Products and Vaccines [n (%)] | 25067 (13.4) | 7998 (9.8) | *0.11* |
| 15: Anaesthesia [n (%)] | 2876 (1.5) | 988 (1.2) | *0.03* |
| 99: Other Preparations, Dressings, Appliances [n (%)] | 25011 (13.3) | 12770 (15.6) | *0.07* |
| Any prescription [n (%)] | 160231 (85.4) | 73284 (89.7) | *0.13* |
| Total prescriptions in 12m before index date [mean (sd)] | 32.5 (51.3) | 33.4 (46.1) | *0.02* |
| **Other treatments** | | | |
| Anxiety & depression treatments [n (%)] | 38857 (20.7) | 19244 (23.6) | *0.07* |
| Anti-hypertensives [n (%)] | 40143 (21.4) | 22037 (27.0) | *0.13* |
| Antidiabetic treatment [n (%)] | 10255 (5.5) | 4408 (5.4) | *<0.01* |

[a]Measurement up to 3 years prior to index date.
[b]Standardised difference

# Table S8: Malignant neoplasm of corpus uteri (ICD-10 C54): characteristics of patients with incident diagnosis in CPRD GOLD and CPRD Aurum

| | Incident cases | | |
|---|---|---|---|
| | CPRD Aurum | CPRD GOLD | *std.diff*[b] |
| **Total patients [n]** | 18424 | 7899 | |
| Age at index date [mean (sd)] | 66.5 (11.6) | 66.8 (11.3) | *0.02* |
| Female [n (%)] | 18424 (100.0) | 7899 (100.0) | -- |
| BMI[a] [mean (sd)] | 29.0 (7.0) | 28.9 (6.6) | *0.01* |
| Missing BMI [n (%)] | 12793 (69.4) | 5511 (69.8) | *0.01* |
| Diastolic blood pressure[a] mmHg [mean (sd)] | 81.3 (10.4) | 80.2 (9.6) | *0.11* |
| Missing diastolic bp [n (%)] | 15824 (85.9) | 7062 (89.4) | *0.11* |
| Systolic blood pressure[a] mmHg [mean (sd)] | 138.8 (20.6) | 135.2 (19.0) | *0.18* |
| Missing systolic bp [n (%)] | 15824 (85.9) | 7062 (89.4) | *0.11* |
| Smoking[a]: current/ex [n (%)] | 10179 (56.7) | 3582 (46.3) | *0.21* |
| Missing smoking status [n (%)] | 487 (2.6) | 159 (2.0) | *0.04* |
| Alcohol status[a]: current/ex [n (%)] | 13022 (77.4) | 5967 (82.3) | *0.12* |
| Missing alcohol status [n (%)] | 1606 (8.7) | 649 (8.2) | *0.02* |
| Years of follow up prior to index date [mean (sd)] | 13.6 (8.5) | 9.5 (6.1) | *0.55* |
| Years of follow up after index date [mean (sd)] | 6.5 (6.2) | 5.1 (4.6) | *0.26* |
| **Healthcare contacts** | | | |
| GP visits in 12m before index date [n (sd)] | 20.3 (15.8) | 14.6 (11.4) | *0.41* |
| GP visits in 12m after index date [n (sd)] | 26.3 (18.5) | 19.0 (14.3) | *0.44* |
| Referrals (definition 1) in 12m before index date [n (sd)] | 0.8 (1.1) | 1.3 (1.4) | *0.42* |
| Referrals (definition 1) in 12m after index date [n (sd)] | 1.5 (1.6) | 1.6 (1.6) | *0.08* |
| Referrals (definition 2) in 12m before index date [n (sd)] | 0.4 (0.9) | 0.6 (1.2) | *0.22* |
| Referrals (definition 2) in 12m after index date [n (sd)] | 0.9 (1.5) | 0.9 (1.6) | *<0.01* |
| **Medical history** | | | |
| Anxiety & depression [n (%)] | 5578 (30.3) | 2601 (32.9) | *0.06* |
| Hypertension [n (%)] | 8283 (45.0) | 3778 (47.8) | *0.06* |
| Cardiovascular disease [n (%)] | 2445 (13.3) | 1117 (14.1) | *0.03* |
| Diabetes [n (%)] | 3022 (16.4) | 1248 (15.8) | *0.02* |
| Chronic respiratory disease [n (%)] | 2483 (13.5) | 1012 (12.8) | *0.02* |
| Chronic kidney disease [n (%)] | 1938 (10.5) | 898 (11.4) | *0.03* |
| Chronic liver disease [n (%)] | 55 (0.3) | 5 (0.1) | *0.06* |
| Alcohol: heavy or harmful use [n (%)] | 403 (2.2) | 187 (2.4) | *0.01* |
| **Treatment history: prescriptions in 12m prior to index date By BNF Chapter** | | | |
| 00: Not classifiable [n (%)] | 3947 (21.4) | 223 (2.8) | *0.59* |
| 01: Gastro-Intestinal System [n (%)] | 6602 (35.8) | 3092 (39.1) | *0.07* |
| 02: Cardiovascular System [n (%)] | 10866 (59.0) | 4975 (63.0) | *0.08* |
| 03: Respiratory System [n (%)] | 3369 (18.3) | 1509 (19.1) | *0.02* |
| 04: Central Nervous System [n (%)] | 9253 (50.2) | 4333 (54.9) | *0.09* |
| 05: Infections [n (%)] | 8249 (44.8) | 3978 (50.4) | *0.11* |
| 06: Endocrine System [n (%)] | 6282 (34.1) | 3021 (38.2) | *0.09* |
| 07: Obstetrics, Gynaecology and Urinary-Tract Disorders [n (%)] | 2331 (12.7) | 1105 (14.0) | *0.04* |
| 08: Malignant Disease and Immunosuppression [n (%)] | 1034 (5.6) | 385 (4.9) | *0.03* |
| 09: Nutrition and Blood [n (%)] | 3647 (19.8) | 1707 (21.6) | *0.04* |
| 10: Musculoskeletal and Joint Diseases [n (%)] | 5168 (28.1) | 2443 (30.9) | *0.06* |
| 11: Eye [n (%)] | 2117 (11.5) | 1013 (12.8) | *0.04* |
| 12: Ear, Nose and Oropharynx [n (%)] | 1817 (9.9) | 901 (11.4) | *0.05* |
| 13: Skin [n (%)] | 4909 (26.6) | 2338 (29.6) | *0.07* |
| 14: Immunological Products and Vaccines [n (%)] | 2972 (16.1) | 815 (10.3) | *0.17* |
| 15: Anaesthesia [n (%)] | 318 (1.7) | 102 (1.3) | *0.04* |
| 99: Other Preparations, Dressings, Appliances [n (%)] | 3414 (18.5) | 1624 (20.6) | *0.05* |
| Any prescription [n (%)] | 16795 (91.2) | 7477 (94.7) | *0.14* |
| Total prescriptions in 12m before index date [mean (sd)] | 39.7 (54.9) | 40.5 (48.7) | *0.02* |
| **Other treatments** | | | |
| Anxiety & depression treatments [n (%)] | 3742 (20.3) | 1821 (23.1) | *0.07* |
| Anti-hypertensives [n (%)] | 6222 (33.8) | 3254 (41.2) | *0.15* |
| Antidiabetic treatment [n (%)] | 2231 (12.1) | 916 (11.6) | *0.02* |

[a]Measurement up to 3 years prior to index date.
[b]Standardised difference

# Table S9: Malignant neoplasm of ovary (ICD-10 C56): characteristics of patients with incident diagnosis in CPRD GOLD and CPRD Aurum

| | Incident cases | | |
|---|---|---|---|
| | CPRD Aurum | CPRD GOLD | *std.diff*[b] |
| **Total patients [n]** | 27360 | 10357 | |
| Age at index date [mean (sd)] | 63.4 (14.7) | 64.6 (14.1) | *0.08* |
| Female [n (%)] | 27360 (100.0) | 10357 (100.0) | -- |
| BMI[a] [mean (sd)] | 26.8 (5.8) | 26.5 (5.2) | *0.05* |
| Missing BMI [n (%)] | 18908 (69.1) | 7237 (69.9) | *0.02* |
| Diastolic blood pressure[a] mmHg [mean (sd)] | 79.4 (10.9) | 79.2 (10.2) | *0.02* |
| Missing diastolic bp [n (%)] | 23023 (84.1) | 8937 (86.3) | *0.06* |
| Systolic blood pressure[a] mmHg [mean (sd)] | 133.4 (21.2) | 132.6 (20.1) | *0.04* |
| Missing systolic bp [n (%)] | 23023 (84.1) | 8937 (86.3) | *0.06* |
| Smoking[a]: current/ex [n (%)] | 15344 (59.1) | 5038 (50.6) | *0.17* |
| Missing smoking status [n (%)] | 1383 (5.1) | 401 (3.9) | *0.06* |
| Alcohol status[a]: current/ex [n (%)] | 18543 (78.0) | 7487 (82.2) | *0.1* |
| Missing alcohol status [n (%)] | 3591 (13.1) | 1247 (12.0) | *0.03* |
| Years of follow up prior to index date [mean (sd)] | 12.3 (8.0) | 8.3 (5.9) | *0.56* |
| Years of follow up after index date [mean (sd)] | 5.0 (5.9) | 3.6 (4.3) | *0.28* |
| **Healthcare contacts** | | | |
| GP visits in 12m before index date [n (sd)] | 19.9 (15.8) | 15.2 (11.6) | *0.34* |
| GP visits in 12m after index date [n (sd)] | 26.4 (21.0) | 19.7 (16.9) | *0.35* |
| Referrals (definition 1) in 12m before index date [n (sd)] | 0.8 (1.1) | 1.4 (1.6) | *0.45* |
| Referrals (definition 1) in 12m after index date [n (sd)] | 1.4 (1.6) | 1.7 (1.8) | *0.16* |
| Referrals (definition 2) in 12m before index date [n (sd)] | 0.4 (1.2) | 0.7 (1.7) | *0.18* |
| Referrals (definition 2) in 12m after index date [n (sd)] | 0.8 (1.7) | 0.9 (1.9) | *0.04* |
| **Medical history** | | | |
| Anxiety & depression [n (%)] | 8223 (30.1) | 3499 (33.8) | *0.08* |
| Hypertension [n (%)] | 9213 (33.7) | 3730 (36.0) | *0.05* |
| Cardiovascular disease [n (%)] | 3243 (11.9) | 1352 (13.1) | *0.04* |
| Diabetes [n (%)] | 2598 (9.5) | 846 (8.2) | *0.05* |
| Chronic respiratory disease [n (%)] | 3845 (14.1) | 1445 (14.0) | *<0.01* |
| Chronic kidney disease [n (%)] | 2060 (7.5) | 773 (7.5) | *<0.01* |
| Chronic liver disease [n (%)] | 93 (0.3) | 7 (0.1) | *0.06* |
| Alcohol: heavy or harmful use [n (%)] | 622 (2.3) | 252 (2.4) | *0.01* |
| **Treatment history: prescriptions in 12m prior to index date By BNF Chapter** | | | |
| 00: Not classifiable [n (%)] | 7348 (26.9) | 540 (5.2) | *0.62* |
| 01: Gastro-Intestinal System [n (%)] | 13105 (47.9) | 5937 (57.3) | *0.19* |
| 02: Cardiovascular System [n (%)] | 13374 (48.9) | 5410 (52.2) | *0.07* |
| 03: Respiratory System [n (%)] | 5497 (20.1) | 2294 (22.1) | *0.05* |
| 04: Central Nervous System [n (%)] | 15282 (55.9) | 6487 (62.6) | *0.14* |
| 05: Infections [n (%)] | 13075 (47.8) | 5555 (53.6) | *0.12* |
| 06: Endocrine System [n (%)] | 8258 (30.2) | 3465 (33.5) | *0.07* |
| 07: Obstetrics, Gynaecology and Urinary-Tract Disorders [n (%)] | 3101 (11.3) | 1219 (11.8) | *0.01* |
| 08: Malignant Disease and Immunosuppression [n (%)] | 905 (3.3) | 250 (2.4) | *0.05* |
| 09: Nutrition and Blood [n (%)] | 5695 (20.8) | 2689 (26.0) | *0.12* |
| 10: Musculoskeletal and Joint Diseases [n (%)] | 7495 (27.4) | 3180 (30.7) | *0.07* |
| 11: Eye [n (%)] | 3043 (11.1) | 1188 (11.5) | *0.01* |
| 12: Ear, Nose and Oropharynx [n (%)] | 2814 (10.3) | 1212 (11.7) | *0.05* |
| 13: Skin [n (%)] | 6867 (25.1) | 2805 (27.1) | *0.05* |
| 14: Immunological Products and Vaccines [n (%)] | 4126 (15.1) | 1116 (10.8) | *0.13* |
| 15: Anaesthesia [n (%)] | 476 (1.7) | 176 (1.7) | *<0.01* |
| 99: Other Preparations, Dressings, Appliances [n (%)] | 4736 (17.3) | 2005 (19.4) | *0.05* |
| Any prescription [n (%)] | 24959 (91.2) | 9845 (95.1) | *0.15* |
| Total prescriptions in 12m before index date [mean (sd)] | 34.3 (49.3) | 34.5 (43.5) | *<0.01* |
| **Other treatments** | | | |
| Anxiety & depression treatments [n (%)] | 5953 (21.8) | 2499 (24.1) | *0.06* |
| Anti-hypertensives [n (%)] | 6784 (24.8) | 3080 (29.7) | *0.11* |
| Antidiabetic treatment [n (%)] | 1883 (6.9) | 619 (6.0) | *0.04* |

[a]Measurement up to 3 years prior to index date.
[b]Standardised difference

## Table S10: Malignant neoplasm of prostate (ICD-10 C61): characteristics of patients with incident diagnosis in CPRD GOLD and CPRD Aurum

| | Incident cases | | |
|---|---|---|---|
| | CPRD Aurum | CPRD GOLD | *std.diff*[b] |
| **Total patients [n]** | 149652 | 63925 | |
| Age at index date [mean (sd)] | 71.9 (9.3) | 71.9 (9.2) | *<0.01* |
| Female [n (%)] | 0 (0) | 0 (0) | -- |
| BMI[a] [mean (sd)] | 26.3 (3.8) | 26.3 (3.8) | *<0.01* |
| Missing BMI [n (%)] | 106086 (70.9) | 44999 (70.4) | *0.01* |
| Diastolic blood pressure[a] mmHg [mean (sd)] | 80.8 (10.1) | 80.2 (9.4) | *0.07* |
| Missing diastolic bp [n (%)] | 132488 (88.5) | 57886 (90.6) | *0.07* |
| Systolic blood pressure[a] mmHg [mean (sd)] | 139.8 (19.5) | 137.5 (17.8) | *0.12* |
| Missing systolic bp [n (%)] | 132488 (88.5) | 57886 (90.6) | *0.07* |
| Smoking[a]: current/ex [n (%)] | 104358 (71.9) | 40218 (64.2) | *0.17* |
| Missing smoking status [n (%)] | 4601 (3.1) | 1271 (2.0) | *0.07* |
| Alcohol status[a]: current/ex [n (%)] | 123495 (90.0) | 54367 (92.0) | *0.07* |
| Missing alcohol status [n (%)] | 12382 (8.3) | 4799 (7.5) | *0.03* |
| Years of follow up prior to index date [mean (sd)] | 13.7 (8.2) | 9.2 (6.1) | *0.63* |
| Years of follow up after index date [mean (sd)] | 5.6 (4.8) | 4.8 (4.1) | *0.18* |
| **Healthcare contacts** | | | |
| GP visits in 12m before index date [n (sd)] | 20.3 (15.4) | 14.5 (11.0) | *0.44* |
| GP visits in 12m after index date [n (sd)] | 27.7 (18.4) | 19.6 (13.7) | *0.5* |
| Referrals (definition 1) in 12m before index date [n (sd)] | 0.8 (1.1) | 1.3 (1.4) | *0.41* |
| Referrals (definition 1) in 12m after index date [n (sd)] | 1.4 (1.6) | 1.6 (1.6) | *0.1* |
| Referrals (definition 2) in 12m before index date [n (sd)] | 0.5 (1.0) | 0.7 (1.4) | *0.22* |
| Referrals (definition 2) in 12m after index date [n (sd)] | 0.9 (1.6) | 0.9 (1.6) | *0.02* |
| **Medical history** | | | |
| Anxiety & depression [n (%)] | 27672 (18.5) | 13035 (20.4) | *0.05* |
| Hypertension [n (%)] | 64808 (43.3) | 28593 (44.7) | *0.03* |
| Cardiovascular disease [n (%)] | 36440 (24.3) | 16285 (25.5) | *0.03* |
| Diabetes [n (%)] | 19311 (12.9) | 7739 (12.1) | *0.02* |
| Chronic respiratory disease [n (%)] | 24460 (16.3) | 10553 (16.5) | *<0.01* |
| Chronic kidney disease [n (%)] | 15162 (10.1) | 6493 (10.2) | *<0.01* |
| Chronic liver disease [n (%)] | 466 (0.3) | 47 (0.1) | *0.05* |
| Alcohol: heavy or harmful use [n (%)] | 7685 (5.1) | 4112 (6.4) | *0.06* |
| **Treatment history: prescriptions in 12m prior to index date By BNF Chapter** | | | |
| 00: Not classifiable [n (%)] | 34847 (23.3) | 2576 (4.0) | *0.58* |
| 01: Gastro-Intestinal System [n (%)] | 59370 (39.7) | 27946 (43.7) | *0.08* |
| 02: Cardiovascular System [n (%)] | 92947 (62.1) | 44734 (70.0) | *0.17* |
| 03: Respiratory System [n (%)] | 30073 (20.1) | 13266 (20.8) | *0.02* |
| 04: Central Nervous System [n (%)] | 64357 (43.0) | 29747 (46.5) | *0.07* |
| 05: Infections [n (%)] | 64673 (43.2) | 30565 (47.8) | *0.09* |
| 06: Endocrine System [n (%)] | 34424 (23.0) | 15519 (24.3) | *0.03* |
| 07: Obstetrics, Gynaecology and Urinary-Tract Disorders [n (%)] | 40047 (26.8) | 10361 (16.2) | *0.26* |
| 08: Malignant Disease and Immunosuppression [n (%)] | 19428 (13.0) | 9453 (14.8) | *0.05* |
| 09: Nutrition and Blood [n (%)] | 19060 (12.7) | 8822 (13.8) | *0.03* |
| 10: Musculoskeletal and Joint Diseases [n (%)] | 44862 (30.0) | 20379 (31.9) | *0.04* |
| 11: Eye [n (%)] | 18824 (12.6) | 8302 (13.0) | *0.01* |
| 12: Ear, Nose and Oropharynx [n (%)] | 19194 (12.8) | 9354 (14.6) | *0.05* |
| 13: Skin [n (%)] | 39480 (26.4) | 18761 (29.3) | *0.07* |
| 14: Immunological Products and Vaccines [n (%)] | 31781 (21.2) | 9010 (14.1) | *0.19* |
| 15: Anaesthesia [n (%)] | 4365 (2.9) | 2066 (3.2) | *0.02* |
| 99: Other Preparations, Dressings, Appliances [n (%)] | 28597 (19.1) | 13343 (20.9) | *0.04* |
| Any prescription [n (%)] | 139910 (93.5) | 61174 (95.7) | *0.1* |
| Total prescriptions in 12m before index date [mean (sd)] | 38.5 (46.7) | 38.9 (42.3) | *0.01* |
| **Other treatments** | | | |
| Anxiety & depression treatments [n (%)] | 18701 (12.5) | 8765 (13.7) | *0.04* |
| Anti-hypertensives [n (%)] | 52356 (35.0) | 25807 (40.4) | *0.11* |
| Antidiabetic treatment [n (%)] | 13694 (9.2) | 5445 (8.5) | *0.02* |

[a]Measurement up to 3 years prior to index date.
[b]Standardised difference

# Table S11: Malignant neoplasm of kidney, except renal pelvis (ICD-10 C64): characteristics of patients with incident diagnosis in CPRD GOLD and CPRD Aurum

| | Incident cases | | |
|---|---|---|---|
| | CPRD Aurum | CPRD GOLD | *std.diff*[b] |
| **Total patients [n]** | 20208 | 5922 | |
| Age at index date [mean (sd)] | 66.3 (15.2) | 66.0 (15.5) | *0.02* |
| Female [n (%)] | 7671 (38.0) | 2265 (38.2) | *0.01* |
| BMI[a] [mean (sd)] | 27.1 (4.9) | 27.1 (4.6) | *0.01* |
| Missing BMI [n (%)] | 15259 (75.5) | 4432 (74.8) | *0.02* |
| Diastolic blood pressure[a] mmHg [mean (sd)] | 79.7 (11.3) | 79.3 (12.1) | *0.04* |
| Missing diastolic bp [n (%)] | 18045 (89.3) | 5360 (90.5) | *0.04* |
| Systolic blood pressure[a] mmHg [mean (sd)] | 134.9 (21.1) | 134.4 (20.8) | *0.03* |
| Missing systolic bp [n (%)] | 18045 (89.3) | 5360 (90.5) | *0.04* |
| Smoking[a]: current/ex [n (%)] | 14096 (72.6) | 3574 (63.4) | *0.2* |
| Missing smoking status [n (%)] | 803 (4.0) | 288 (4.9) | *0.04* |
| Alcohol status[a]: current/ex [n (%)] | 15279 (84.4) | 4522 (86.2) | *0.05* |
| Missing alcohol status [n (%)] | 2109 (10.4) | 679 (11.5) | *0.03* |
| Years of follow up prior to index date [mean (sd)] | 13.4 (8.5) | 8.8 (6.1) | *0.63* |
| Years of follow up after index date [mean (sd)] | 4.4 (5.1) | 3.8 (4.3) | *0.13* |
| **Healthcare contacts** | | | |
| GP visits in 12m before index date [n (sd)] | 23.9 (17.3) | 17.1 (12.7) | *0.45* |
| GP visits in 12m after index date [n (sd)] | 29.0 (20.4) | 20.2 (16.3) | *0.47* |
| Referrals (definition 1) in 12m before index date [n (sd)] | 0.8 (1.2) | 1.5 (1.8) | *0.44* |
| Referrals (definition 1) in 12m after index date [n (sd)] | 1.5 (1.7) | 1.8 (2.0) | *0.14* |
| Referrals (definition 2) in 12m before index date [n (sd)] | 0.5 (1.1) | 0.8 (1.6) | *0.24* |
| Referrals (definition 2) in 12m after index date [n (sd)] | 1.0 (1.7) | 1.1 (1.9) | *0.04* |
| **Medical history** | | | |
| Anxiety & depression [n (%)] | 5567 (27.5) | 1628 (27.5) | *<0.01* |
| Hypertension [n (%)] | 10094 (50.0) | 2936 (49.6) | *0.01* |
| Cardiovascular disease [n (%)] | 4672 (23.1) | 1398 (23.6) | *0.01* |
| Diabetes [n (%)] | 3418 (16.9) | 873 (14.7) | *0.06* |
| Chronic respiratory disease [n (%)] | 3633 (18.0) | 964 (16.3) | *0.05* |
| Chronic kidney disease [n (%)] | 3251 (16.1) | 964 (16.3) | *0.01* |
| Chronic liver disease [n (%)] | 151 (0.7) | 8 (0.1) | *0.09* |
| Alcohol: heavy or harmful use [n (%)] | 1051 (5.2) | 357 (6.0) | *0.04* |
| **Treatment history: prescriptions in 12m prior to index date By BNF Chapter** | | | |
| 00: Not classifiable [n (%)] | 5218 (25.8) | 293 (4.9) | *0.6* |
| 01: Gastro-Intestinal System [n (%)] | 10085 (49.9) | 3219 (54.4) | *0.09* |
| 02: Cardiovascular System [n (%)] | 13030 (64.5) | 3933 (66.4) | *0.04* |
| 03: Respiratory System [n (%)] | 4986 (24.7) | 1511 (25.5) | *0.02* |
| 04: Central Nervous System [n (%)] | 11659 (57.7) | 3697 (62.4) | *0.1* |
| 05: Infections [n (%)] | 10626 (52.6) | 3380 (57.1) | *0.09* |
| 06: Endocrine System [n (%)] | 6350 (31.4) | 1867 (31.5) | *<0.01* |
| 07: Obstetrics, Gynaecology and Urinary-Tract Disorders [n (%)] | 2948 (14.6) | 645 (10.9) | *0.11* |
| 08: Malignant Disease and Immunosuppression [n (%)] | 678 (3.4) | 222 (3.7) | *0.02* |
| 09: Nutrition and Blood [n (%)] | 4965 (24.6) | 1586 (26.8) | *0.05* |
| 10: Musculoskeletal and Joint Diseases [n (%)] | 6497 (32.2) | 2025 (34.2) | *0.04* |
| 11: Eye [n (%)] | 2459 (12.2) | 771 (13.0) | *0.03* |
| 12: Ear, Nose and Oropharynx [n (%)] | 2597 (12.9) | 823 (13.9) | *0.03* |
| 13: Skin [n (%)] | 5524 (27.3) | 1734 (29.3) | *0.04* |
| 14: Immunological Products and Vaccines [n (%)] | 3849 (19.0) | 756 (12.8) | *0.17* |
| 15: Anaesthesia [n (%)] | 491 (2.4) | 156 (2.6) | *0.01* |
| 99: Other Preparations, Dressings, Appliances [n (%)] | 4438 (22.0) | 1348 (22.8) | *0.02* |
| Any prescription [n (%)] | 18962 (93.8) | 5707 (96.4) | *0.12* |
| Total prescriptions in 12m before index date [mean (sd)] | 48.7 (63.4) | 44.6 (48.0) | *0.07* |
| **Other treatments** | | | |
| Anxiety & depression treatments [n (%)] | 4265 (21.1) | 1332 (22.5) | *0.03* |
| Anti-hypertensives [n (%)] | 8401 (41.6) | 2694 (45.5) | *0.08* |
| Antidiabetic treatment [n (%)] | 2532 (12.5) | 651 (11.0) | *0.05* |

[a]Measurement up to 3 years prior to index date.
[b]Standardised difference

# Table S12: Malignant neoplasm of bladder (ICD-10 C67): characteristics of patients with incident diagnosis in CPRD GOLD and CPRD Aurum

| | Incident cases | | |
|---|---|---|---|
| | CPRD Aurum | CPRD GOLD | *std.diff*[b] |
| **Total patients [n]** | 51192 | 21369 | |
| Age at index date [mean (sd)] | 72.2 (11.4) | 72.5 (11.2) | *0.03* |
| Female [n (%)] | 13416 (26.2) | 5796 (27.1) | *0.02* |
| BMI[a] [mean (sd)] | 26.4 (4.5) | 26.2 (4.6) | *0.03* |
| Missing BMI [n (%)] | 36500 (71.3) | 15464 (72.4) | *0.02* |
| Diastolic blood pressure[a] mmHg [mean (sd)] | 80.9 (10.5) | 80.4 (9.8) | *0.05* |
| Missing diastolic bp [n (%)] | 44502 (86.9) | 19197 (89.8) | *0.09* |
| Systolic blood pressure[a] mmHg [mean (sd)] | 141.1 (21.0) | 138.5 (19.9) | *0.12* |
| Missing systolic bp [n (%)] | 44502 (86.9) | 19197 (89.8) | *0.09* |
| Smoking[a]: current/ex [n (%)] | 38589 (78.4) | 15109 (73.0) | *0.13* |
| Missing smoking status [n (%)] | 1979 (3.9) | 666 (3.1) | *0.04* |
| Alcohol status[a]: current/ex [n (%)] | 39529 (85.8) | 17070 (88.5) | *0.08* |
| Missing alcohol status [n (%)] | 5144 (10.0) | 2091 (9.8) | *0.01* |
| Years of follow up prior to index date [mean (sd)] | 13.1 (8.3) | 8.8 (6.0) | *0.59* |
| Years of follow up after index date [mean (sd)] | 5.6 (5.6) | 4.5 (4.5) | *0.22* |
| **Healthcare contacts** | | | |
| GP visits in 12m before index date [n (sd)] | 21.9 (17.7) | 16.0 (12.6) | *0.39* |
| GP visits in 12m after index date [n (sd)] | 27.4 (20.6) | 19.2 (15.7) | *0.45* |
| Referrals (definition 1) in 12m before index date [n (sd)] | 0.8 (1.2) | 1.4 (1.6) | *0.42* |
| Referrals (definition 1) in 12m after index date [n (sd)] | 1.5 (1.7) | 1.7 (1.8) | *0.12* |
| Referrals (definition 2) in 12m before index date [n (sd)] | 0.5 (1.1) | 0.8 (1.5) | *0.21* |
| Referrals (definition 2) in 12m after index date [n (sd)] | 1.0 (1.7) | 1.0 (1.8) | *0.02* |
| **Medical history** | | | |
| Anxiety & depression [n (%)] | 11501 (22.5) | 5194 (24.3) | *0.04* |
| Hypertension [n (%)] | 22458 (43.9) | 9656 (45.2) | *0.03* |
| Cardiovascular disease [n (%)] | 14326 (28.0) | 6287 (29.4) | *0.03* |
| Diabetes [n (%)] | 7965 (15.6) | 3192 (14.9) | *0.02* |
| Chronic respiratory disease [n (%)] | 9392 (18.3) | 3883 (18.2) | *<0.01* |
| Chronic kidney disease [n (%)] | 7008 (13.7) | 3016 (14.1) | *0.01* |
| Chronic liver disease [n (%)] | 255 (0.5) | 19 (0.1) | *0.08* |
| Alcohol: heavy or harmful use [n (%)] | 2472 (4.8) | 1249 (5.8) | *0.05* |
| **Treatment history: prescriptions in 12m prior to index date By BNF Chapter** | | | |
| 00: Not classifiable [n (%)] | 13328 (26.0) | 986 (4.6) | *0.62* |
| 01: Gastro-Intestinal System [n (%)] | 21113 (41.2) | 9808 (45.9) | *0.09* |
| 02: Cardiovascular System [n (%)] | 32405 (63.3) | 14589 (68.3) | *0.1* |
| 03: Respiratory System [n (%)] | 11488 (22.4) | 5055 (23.7) | *0.03* |
| 04: Central Nervous System [n (%)] | 25310 (49.4) | 11662 (54.6) | *0.1* |
| 05: Infections [n (%)] | 30926 (60.4) | 14375 (67.3) | *0.14* |
| 06: Endocrine System [n (%)] | 14758 (28.8) | 6383 (29.9) | *0.02* |
| 07: Obstetrics, Gynaecology and Urinary-Tract Disorders [n (%)] | 10175 (19.9) | 3352 (15.7) | *0.11* |
| 08: Malignant Disease and Immunosuppression [n (%)] | 1349 (2.6) | 524 (2.5) | *0.01* |
| 09: Nutrition and Blood [n (%)] | 9462 (18.5) | 4486 (21.0) | *0.06* |
| 10: Musculoskeletal and Joint Diseases [n (%)] | 14128 (27.6) | 6169 (28.9) | *0.03* |
| 11: Eye [n (%)] | 6649 (13.0) | 2988 (14.0) | *0.03* |
| 12: Ear, Nose and Oropharynx [n (%)] | 6047 (11.8) | 2930 (13.7) | *0.06* |
| 13: Skin [n (%)] | 14318 (28.0) | 6663 (31.2) | *0.07* |
| 14: Immunological Products and Vaccines [n (%)] | 10330 (20.2) | 2903 (13.6) | *0.18* |
| 15: Anaesthesia [n (%)] | 1132 (2.2) | 533 (2.5) | *0.02* |
| 99: Other Preparations, Dressings, Appliances [n (%)] | 11076 (21.6) | 4905 (23.0) | *0.03* |
| Any prescription [n (%)] | 47344 (92.5) | 20528 (96.1) | *0.15* |
| Total prescriptions in 12m before index date [mean (sd)] | 46.8 (56.9) | 45.5 (49.0) | *0.02* |
| **Other treatments** | | | |
| Anxiety & depression treatments [n (%)] | 8195 (16.0) | 3775 (17.7) | *0.04* |
| Anti-hypertensives [n (%)] | 18449 (36.0) | 8952 (41.9) | *0.12* |
| Antidiabetic treatment [n (%)] | 5733 (11.2) | 2278 (10.7) | *0.02* |

[a]Measurement up to 3 years prior to index date.
[b]Standardised difference

# Table S13: Non-Hodgkin's lymphoma (ICD-10 C82-C85): characteristics of patients with incident diagnosis in CPRD GOLD and CPRD Aurum

| | Incident cases | | |
|---|---|---|---|
| | CPRD Aurum | CPRD GOLD | *std.diff*[b] |
| **Total patients [n]** | 45955 | 19352 | |
| Age at index date [mean (sd)] | 64.0 (17.3) | 65.5 (16.1) | *0.09* |
| Female [n (%)] | 20821 (45.3) | 9013 (46.6) | *0.03* |
| BMI[a] [mean (sd)] | 26.2 (4.8) | 26.3 (4.6) | *0.01* |
| Missing BMI [n (%)] | 32508 (70.7) | 13738 (71.0) | *0.01* |
| Diastolic blood pressure[a] mmHg [mean (sd)] | 78.1 (10.6) | 78.2 (9.9) | *0.01* |
| Missing diastolic bp [n (%)] | 38696 (84.2) | 16685 (86.2) | *0.06* |
| Systolic blood pressure[a] mmHg [mean (sd)] | 131.5 (19.3) | 132.3 (18.9) | *0.04* |
| Missing systolic bp [n (%)] | 38696 (84.2) | 16685 (86.2) | *0.06* |
| Smoking[a]: current/ex [n (%)] | 29085 (66.2) | 10878 (58.4) | *0.16* |
| Missing smoking status [n (%)] | 2001 (4.4) | 735 (3.8) | *0.03* |
| Alcohol status[a]: current/ex [n (%)] | 33416 (82.4) | 14817 (86.5) | *0.11* |
| Missing alcohol status [n (%)] | 5415 (11.8) | 2216 (11.5) | *0.01* |
| Years of follow up prior to index date [mean (sd)] | 12.6 (8.3) | 8.5 (6.0) | *0.56* |
| Years of follow up after index date [mean (sd)] | 5.2 (5.4) | 4.4 (4.4) | *0.17* |
| **Healthcare contacts** | | | |
| GP visits in 12m before index date [n (sd)] | 22.0 (17.7) | 16.0 (12.6) | *0.39* |
| GP visits in 12m after index date [n (sd)] | 27.0 (20.5) | 19.0 (16.1) | *0.43* |
| Referrals (definition 1) in 12m before index date [n (sd)] | 0.8 (1.2) | 1.4 (1.7) | *0.39* |
| Referrals (definition 1) in 12m after index date [n (sd)] | 1.4 (1.7) | 1.6 (1.8) | *0.12* |
| Referrals (definition 2) in 12m before index date [n (sd)] | 0.4 (1.1) | 0.6 (1.5) | *0.17* |
| Referrals (definition 2) in 12m after index date [n (sd)] | 0.8 (1.6) | 0.8 (1.7) | *0.01* |
| **Medical history** | | | |
| Anxiety & depression [n (%)] | 11283 (24.6) | 5109 (26.4) | *0.04* |
| Hypertension [n (%)] | 15449 (33.6) | 6836 (35.3) | *0.04* |
| Cardiovascular disease [n (%)] | 8227 (17.9) | 3784 (19.6) | *0.04* |
| Diabetes [n (%)] | 5145 (11.2) | 2072 (10.7) | *0.02* |
| Chronic respiratory disease [n (%)] | 7341 (16.0) | 3220 (16.6) | *0.02* |
| Chronic kidney disease [n (%)] | 4642 (10.1) | 1931 (10.0) | *<0.01* |
| Chronic liver disease [n (%)] | 380 (0.8) | 8 (0.0) | *0.12* |
| Alcohol: heavy or harmful use [n (%)] | 1750 (3.8) | 769 (4.0) | *0.01* |
| **Treatment history: prescriptions in 12m prior to index date By BNF Chapter** | | | |
| 00: Not classifiable [n (%)] | 11873 (25.8) | 827 (4.3) | *0.63* |
| 01: Gastro-Intestinal System [n (%)] | 20265 (44.1) | 9654 (49.9) | *0.12* |
| 02: Cardiovascular System [n (%)] | 22801 (49.6) | 10448 (54.0) | *0.09* |
| 03: Respiratory System [n (%)] | 11227 (24.4) | 5192 (26.8) | *0.05* |
| 04: Central Nervous System [n (%)] | 24071 (52.4) | 11165 (57.7) | *0.11* |
| 05: Infections [n (%)] | 23808 (51.8) | 10983 (56.8) | *0.1* |
| 06: Endocrine System [n (%)] | 12899 (28.1) | 5933 (30.7) | *0.06* |
| 07: Obstetrics, Gynaecology and Urinary-Tract Disorders [n (%)] | 5318 (11.6) | 1768 (9.1) | *0.08* |
| 08: Malignant Disease and Immunosuppression [n (%)] | 1347 (2.9) | 518 (2.7) | *0.02* |
| 09: Nutrition and Blood [n (%)] | 9928 (21.6) | 4776 (24.7) | *0.07* |
| 10: Musculoskeletal and Joint Diseases [n (%)] | 13555 (29.5) | 6454 (33.4) | *0.08* |
| 11: Eye [n (%)] | 6327 (13.8) | 2850 (14.7) | *0.03* |
| 12: Ear, Nose and Oropharynx [n (%)] | 6685 (14.5) | 3283 (17.0) | *0.07* |
| 13: Skin [n (%)] | 14068 (30.6) | 6524 (33.7) | *0.07* |
| 14: Immunological Products and Vaccines [n (%)] | 7962 (17.3) | 2245 (11.6) | *0.16* |
| 15: Anaesthesia [n (%)] | 940 (2.0) | 356 (1.8) | *0.01* |
| 99: Other Preparations, Dressings, Appliances [n (%)] | 9407 (20.5) | 4254 (22.0) | *0.04* |
| Any prescription [n (%)] | 41921 (91.2) | 18331 (94.7) | *0.14* |
| Total prescriptions in 12m before index date [mean (sd)] | 38.5 (54.1) | 38.7 (45.5) | *<0.01* |
| **Other treatments** | | | |
| Anxiety & depression treatments [n (%)] | 8854 (19.3) | 4046 (20.9) | *0.04* |
| Anti-hypertensives [n (%)] | 12530 (27.3) | 6183 (32.0) | *0.1* |
| Antidiabetic treatment [n (%)] | 3699 (8.0) | 1519 (7.8) | *0.01* |

[a]Measurement up to 3 years prior to index date.
[b]Standardised difference

# Table S14: Leukaemia (ICD-10 C91-C95): characteristics of patients with incident diagnosis in CPRD GOLD and CPRD Aurum

| | Incident cases | | |
|---|---|---|---|
| | CPRD Aurum | CPRD GOLD | *std.diff*[b] |
| **Total patients [n]** | 36476 | 16155 | |
| Age at index date [mean (sd)] | 65.4 (19.6) | 65.8 (19.4) | *0.02* |
| Female [n (%)] | 15299 (41.9) | 6778 (42.0) | *<0.01* |
| BMI[a] [mean (sd)] | 26.3 (4.7) | 26.4 (4.5) | *0.02* |
| Missing BMI [n (%)] | 26095 (71.5) | 11606 (71.8) | *0.01* |
| Diastolic blood pressure[a] mmHg [mean (sd)] | 77.5 (11.2) | 77.0 (10.7) | *0.04* |
| Missing diastolic bp [n (%)] | 31068 (85.2) | 14098 (87.3) | *0.06* |
| Systolic blood pressure[a] mmHg [mean (sd)] | 132.3 (21.3) | 131.3 (19.8) | *0.05* |
| Missing systolic bp [n (%)] | 31068 (85.2) | 14098 (87.3) | *0.06* |
| Smoking[a]: current/ex [n (%)] | 22304 (66.4) | 8866 (58.9) | *0.15* |
| Missing smoking status [n (%)] | 2872 (7.9) | 1115 (6.9) | *0.04* |
| Alcohol status[a]: current/ex [n (%)] | 25346 (83.1) | 11840 (86.5) | *0.09* |
| Missing alcohol status [n (%)] | 5971 (16.4) | 2466 (15.3) | *0.03* |
| Years of follow up prior to index date [mean (sd)] | 12.3 (8.2) | 8.3 (6.1) | *0.55* |
| Years of follow up after index date [mean (sd)] | 5.0 (5.4) | 4.1 (4.4) | *0.18* |
| **Healthcare contacts** | | | |
| GP visits in 12m before index date [n (sd)] | 19.8 (17.4) | 14.3 (12.5) | *0.36* |
| GP visits in 12m after index date [n (sd)] | 23.5 (19.0) | 16.0 (14.8) | *0.44* |
| Referrals (definition 1) in 12m before index date [n (sd)] | 0.7 (1.1) | 1.1 (1.6) | *0.34* |
| Referrals (definition 1) in 12m after index date [n (sd)] | 1.2 (1.7) | 1.4 (1.8) | *0.1* |
| Referrals (definition 2) in 12m before index date [n (sd)] | 0.5 (1.3) | 0.7 (1.7) | *0.17* |
| Referrals (definition 2) in 12m after index date [n (sd)] | 0.9 (1.8) | 0.9 (1.9) | *0.02* |
| **Medical history** | | | |
| Anxiety & depression [n (%)] | 8151 (22.3) | 3929 (24.3) | *0.05* |
| Hypertension [n (%)] | 13016 (35.7) | 6155 (38.1) | *0.05* |
| Cardiovascular disease [n (%)] | 7514 (20.6) | 3572 (22.1) | *0.04* |
| Diabetes [n (%)] | 4392 (12.0) | 1867 (11.6) | *0.02* |
| Chronic respiratory disease [n (%)] | 5795 (15.9) | 2509 (15.5) | *0.01* |
| Chronic kidney disease [n (%)] | 3498 (9.6) | 1606 (9.9) | *0.01* |
| Chronic liver disease [n (%)] | 165 (0.5) | 8 (0.0) | *0.08* |
| Alcohol: heavy or harmful use [n (%)] | 1339 (3.7) | 694 (4.3) | *0.03* |
| **Treatment history: prescriptions in 12m prior to index date**<br>**By BNF Chapter** | | | |
| 00: Not classifiable [n (%)] | 8956 (24.6) | 694 (4.3) | *0.6* |
| 01: Gastro-Intestinal System [n (%)] | 13688 (37.5) | 6723 (41.6) | *0.08* |
| 02: Cardiovascular System [n (%)] | 19271 (52.8) | 9166 (56.7) | *0.08* |
| 03: Respiratory System [n (%)] | 8387 (23.0) | 3890 (24.1) | *0.03* |
| 04: Central Nervous System [n (%)] | 17308 (47.5) | 8418 (52.1) | *0.09* |
| 05: Infections [n (%)] | 17913 (49.1) | 8656 (53.6) | *0.09* |
| 06: Endocrine System [n (%)] | 9782 (26.8) | 4585 (28.4) | *0.03* |
| 07: Obstetrics, Gynaecology and Urinary-Tract Disorders [n (%)] | 4123 (11.3) | 1440 (8.9) | *0.08* |
| 08: Malignant Disease and Immunosuppression [n (%)] | 1293 (3.5) | 537 (3.3) | *0.01* |
| 09: Nutrition and Blood [n (%)] | 7194 (19.7) | 3484 (21.6) | *0.05* |
| 10: Musculoskeletal and Joint Diseases [n (%)] | 10408 (28.5) | 5049 (31.3) | *0.06* |
| 11: Eye [n (%)] | 5110 (14.0) | 2388 (14.8) | *0.02* |
| 12: Ear, Nose and Oropharynx [n (%)] | 5105 (14.0) | 2635 (16.3) | *0.06* |
| 13: Skin [n (%)] | 10298 (28.2) | 4942 (30.6) | *0.05* |
| 14: Immunological Products and Vaccines [n (%)] | 6662 (18.3) | 1946 (12.0) | *0.17* |
| 15: Anaesthesia [n (%)] | 843 (2.3) | 346 (2.1) | *0.01* |
| 99: Other Preparations, Dressings, Appliances [n (%)] | 6621 (18.2) | 3260 (20.2) | *0.05* |
| Any prescription [n (%)] | 32988 (90.4) | 15136 (93.7) | *0.12* |
| Total prescriptions in 12m before index date [mean (sd)] | 39.2 (58.2) | 39.3 (48.2) | *<0.01* |
| **Other treatments** | | | |
| Anxiety & depression treatments [n (%)] | 6036 (16.5) | 2843 (17.6) | *0.03* |
| Anti-hypertensives [n (%)] | 10423 (28.6) | 5520 (34.2) | *0.12* |
| Antidiabetic treatment [n (%)] | 3116 (8.5) | 1357 (8.4) | *0.01* |

[a]Measurement up to 3 years prior to index date.
[b]Standardised difference

Figure S2: Boxplot summary of standardised differences in incident HD and cancer patients in CPRD GOLD and CPRD Aurum, for different types* of patient characteristic.

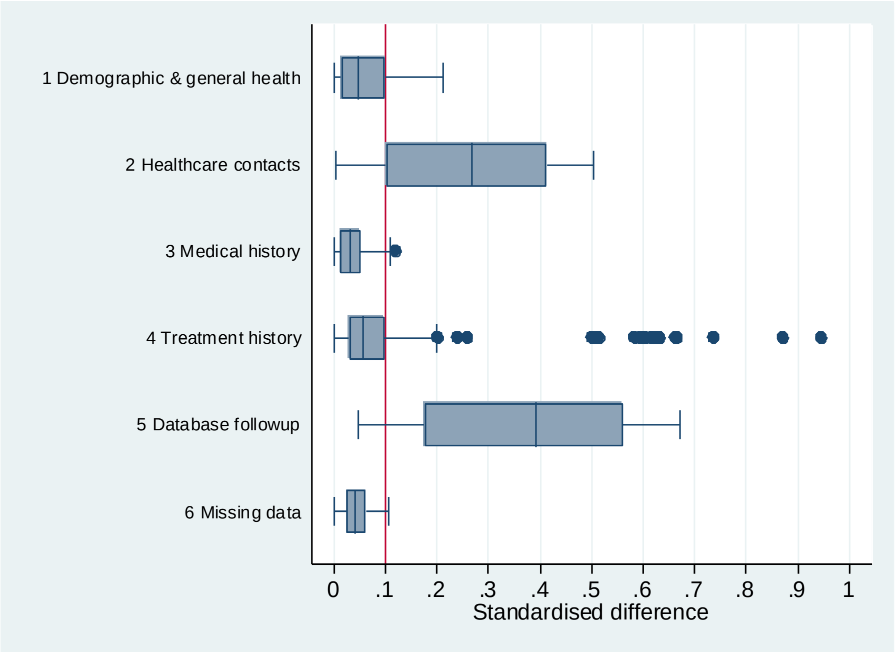


* see Methods section for details of types of characteristic.

## Appendix 1: Codelists

| Codelist description | CPRD Aurum filename | Aurum Reference | CPRD GOLD filename | GOLD Reference |
|---|---|---|---|---|
| Huntington's Disease | HuntingtonsDisease_Aurum.txt | | HuntingtonsDisease_GOLD.txt | |
| Smoking | Smoking_Aurum.txt | [a] | Smoking_GOLD.txt | [a] |
| Alcohol Consumption | AlcoholConsumption_Aurum.txt | -- | AlcoholConsumption_GOLD.txt | -- |
| Height Weight BMI | HeightWeightBMI_Aurum.txt | [a] | n/a | |
| Anxiety Depression | AnxietyDepression_Aurum.txt | -- | AnxietyDepression_GOLD.txt | [b] |
| Hypertension | Hypertension_Aurum.txt | [a] | Hypertension_GOLD.txt | [a] |
| Blood pressure | BloodPressure_Aurum.txt | -- | n/a | |
| Cardiovascular Disease | CardiovascularDisease_Aurum.txt | [a] | CardiovascularDisease_GOLD.txt | [a] |
| Diabetes | Diabetes_Aurum.txt | [a] | Diabetes_GOLD.txt | [a] |
| Chronic Respiratory Disease | ChronicRespiratoryDisease_Aurum.txt | [a] | ChronicRespiratoryDisease_GOLD.txt | [a] |
| Chronic Kidney Disease | ChronicKidneyDisease_Aurum.txt | [a] | ChronicKidneyDisease_GOLD.txt | [a] |
| Chronic Liver Disease | ChronicLiverDisease_Aurum.txt | [a] | ChronicLiverDisease_GOLD.txt | [a] |
| Alcohol Harmful Use | AlcoholHarmfulUse_Aurum.txt | [c] | AlcoholHarmfulUse_GOLD.txt | [c] |
| Referral Medcodes | ReferralMedcodes_Aurum.txt | -- | ReferralMedcodes_GOLD.txt | -- |
| Smoking Cessation Treatment | SmokingCessationTreatment_Aurum.txt | -- | SmokingCessationTreatment_GOLD.txt | -- |
| Alcohol Cessation Treatment | AlcoholCessationTreatment_Aurum.txt | -- | AlcoholCessationTreatment_GOLD.txt | -- |
| Anxiety Depression Treatment | AnxietyDepressionTreatment_Aurum.txt | | AnxietyDepressionTreatment_GOLD.txt | [b] |
| Antihypertensive Treatment | AntihypertensiveTreatment_Aurum.txt | [d] | AntihypertensiveTreatment_GOLD.txt | [d] |
| Tetrabenazine Treatment | TetrabenazineTreatment_Aurum.txt | -- | TetrabenazineTreatment_GOLD.txt | -- |
| Antidiabetic Treatment | AntidiabeticTreatment_Aurum.txt | [d] | AntidiabeticTreatment_GOLD.txt | [d] |
| Cancer: 14 Sites | Cancer14Sites_Aurum.txt | -- | Cancer14Sites_GOLD.txt | [e] |
| Cancer: all sites | CancerAllSites_Aurum.txt | -- | CancerAllSites_GOLD.txt | [e] |
| | | | | |
| | ICD-9 codes | | ICD-10 codes | |
| Cancer 14 Sites: ICD-9 \ ICD-10 codes | Cancer14Sites_ICD9.txt | [e] | Cancer14Sites_ICD10.txt | [e] |

[a] Davidson J, Warren-Gash C, Mcdonald H, Banerjee A, Gayle A, Strongman H, Evans D, Clay S, Forbes H, Mansfield KE, Carreira H, Bhaskaran K, Bhate K, Smeeth L, Dedman D, Mathur R, Cadogan S and LSHTM EHR team. Codelists for: *"Factors associated with excess mortality in the first wave of COVID-19 pandemic in the UK: a cohort analysis using the Clinical Practice Research Databank"* [Internet]. London School of Hygiene & Tropical Medicine; 2021. Available from: https://doi.org/10.17037/DATA.00002269.

[b] Muzambi R, Bhaskaran K, Smeeth L and Warren-Gash C (2021). Codelists for: *"Common infections and incident dementia: a historical cohort study using UK primary and secondary care data"*. [Project]. London School of Hygiene & Tropical Medicine, London, United Kingdom. https://doi.org/10.17037/DATA.00002073.

[c] Schonmann Y, Mansfield KE, Hayes J, Roberts A, Smeeth L and Langan SM (2018). Code lists for: *"Atopic Eczema in Adulthood and Risk of Depression and Anxiety: A Population-Based Cohort Study"*. [Project]. London School of Hygiene & Tropical Medicine, London, United Kingdom. https://doi.org/10.17037/DATA.00000941.

[d] Davidson J, Warren-Gash C, Mcdonald H, Banerjee A, Smeeth L and Strongman H (2021). Codelists for: *"Underlying cardiovascular risk and major adverse cardiovascular events after acute respiratory infection: a population-based cohort study of over 4.2 million individuals in England, 2008-2018"*. [Project]. London School of Hygiene & Tropical Medicine, London, United Kingdom. https://doi.org/10.17037/DATA.00002240.

[e] Bhaskaran K, Douglas I, Forbes H, dos-Santos-Silva I, Leon DA, Smeeth L. *Body-mass index and risk of 22 specific cancers: a population-based cohort study of 5·24 million UK adults*. Lancet. 2014 Aug 30;384(9945):755-65. https://doi.org/10.1016/S0140-6736(14)60892-8

[e] Strongman H, Williams R, Bhaskaran K. What are the implications of using individual and combined sources of routinely collected data to identify and characterise incident site-specific cancers? a concordance and validation study using linked English electronic health records data. BMJ open. 2020;10:e037719. https://doi.org/10.1136/bmjopen-2020-037719

Codelists are available as separate files in the supplementary materials. See methods section for more information on creating codelists *de novo*.
