# Supplementary material for: Pooling of primary care electronic health record (EHR) data on Huntington’s disease (HD) and cancer: establishing comparability of two large UK databases
Source: BMJ Open. 2024 Feb 14;14(2):e070258. doi: 10.1136/bmjopen-2022-070258 (PMC10868307; doi:10.1136/bmjopen-2022-070258)
Supplement: Supplementary file 2 [file bmjopen-2022-070258.reviewer_comments.pdf]

PEER REVIEW HISTORY

BMJ Open publishes all reviews undertaken for accepted manuscripts. Reviewers are asked to complete a checklist review form (**http://bmjopen.bmj.com/site/about/resources/checklist.pdf**) and are provided with free text boxes to elaborate on their assessment. These free text comments are reproduced below.

ARTICLE DETAILS

**TITLE (PROVISIONAL)**	Pooling of primary care electronic health record (EHR) data on Huntington's disease (HD) and cancer: establishing comparability of two large UK databases
**AUTHORS**	Dedman, Daniel; Williams, Rachael; Bhaskaran, Krishnan; Douglas, Ian

VERSION 1 – REVIEW

**REVIEWER**	Persephone Doupi<br>National Institute for Health and Welfare, Information Department
**REVIEW RETURNED**	18-Jul-2023

**GENERAL COMMENTS**	Review bmjopen-2022-070258<br>The study presents the comparison of baseline characteristics of patient populations in two UK primary databases concerning Huntingdon's disease (HD - a rare condition) and 14 different cancers (10 of the most common types included). The comparison is used as a means of proving feasibility of using data pooled from the two databases, in order to increase statistical power for further studies. The work presented was undertaken in order ”to inform a planned investigation into cancer risks among people with HD.” (p.6, lines 47-48).<br>The general topic of large database utilization in observational studies is very topical and even more so when it comes to the more scarcely available and very valuable primary care data, as in the case of this particular study.<br>My recommendation would be to publish the paper with certain revisions, which could be considered minor, even though in my view they have a significant impact in how the study is perceived and interpreted.<br>The study itself and its presentation in the manuscript are for the most part rather straightforward, clear and well executed.<br>I did struggle with a few issues though:<br>- What should the reader expect of the paper based on its title?<br>- What are the implications of the approach used from the viewpoint of observational study adoption and related requirements?<br>- In conjunction with the previous question, do the findings presented by the study team support their conclusions?<br>Starting with the title: I think that the current title, ”Feasibility of pooling patient data from multiple electronic health record (EHR) databases: Case studies in UK primary care”, to some extent creates too big expectations to the reader and does not really reflect the content of the work presented.<br>I could not understand the use of the term ”case study” and even more so in plural, in the specific context.<br>Moreover, the level of assessing feasibility of pooling patient data

	based on the approach and findings of the study implies a very specific and (in my view) rather narrow understanding of feasibility. The comparison undertaken by the study team is indeed the first step in the pathway of utilizing combined patient data, (p5, Introduction -lines 22-23). However, there are still many unanswered questions and additional work ahead, particularly with regard to various aspects of data quality. The aforementioned further steps may eventually render the combination of data either too complex or non-suitable for certain types or whole areas of research questions. Should we in these cases still say that pooling of data is feasible?<br>I would hence favour a more straightforward approach to the title, providing more specific details of the study content, such as:<br>“Pooling of primary care Electronic Health Record data on HD and cancers: establishing comparability of two large UK databases” or “Combining primary care Electronic Health Record data on HD and cancers: a descriptive study of two large UK databases”<br>Moving to the other areas of concern, namely the implications of the study for observational health research studies and the conclusions drawn by the authors.<br>The authors clearly describe the sources of challenges in combining data from the two databases: Even though the “databases are similar in terms of source population and setting, each database is derived from a different GP software system employing different clinical dictionaries and coding systems, user interface and data capture methods, and data structures.”<br>However, the information provided to the reader with regard to the methodology and the results of code harmonisation work undertaken by the study team is rather limited:<br>Appendix 1 on page 43 is not really useful for the reviewer/reader since the actually used codings in the two databases are not accessible – only .txt files are visible in the pdf version. This shortcoming does not allow for assessment of the quality of the mappings and choices made by the study team in code harmonisation between the two databases, which is a key issue in EHR-data research use (see e.g. Williams et al. Clinical code set engineering for reusing EHR data for research: A review. http://dx.doi.org/10.1016/j.jbi.2017.04.010).<br>In addition to more methodological details, also a more specific list of the codelists utilized could be provided in the references instead of the general entry web page of the Data Compass e.g. a) Codelists for: "Risk of fractures in half a million survivors of 20 cancers: a population-based matched cohort study using linked UK electronic health records"<br>or b) Code lists used in "Medium and long-term risks of specific cardiovascular diseases among 108,215 survivors of 20 adult cancers: population-based cohort study using multiple linked UK electronic health records databases" (Accessed and selected as possible examples at: https://datacompass.lshtm.ac.uk/view/types/collection/index.C.html)<br>In addition to the limited information provided as to how data quality issues resulting from differences in coding practices and data models underlying the source systems feeding to the two databases were resolved by the study team, the weight of these challenges in regard to combining patient data is in my view underplayed by the authors.<br>The section of Research in content presents a very good overview of all the sources of variability:<br>”regional differences and their changes overtime, differences in data curation and – most crucially – differences related to the GP software used for documentation: differences in user interface and

	how this supports recording of accurately coded clinical information, different clinical and drug dictionaries and coding systems, as well as differences in the underlying data models used to represent and store data"<br>Even though the authors have recognized the sources of problems in data pooling, in the Conclusions section of the paper they skip discussing the impact of these problems on the actual pooling process and analysis phases and only recommend caution in the interpretation of results.<br>"As well as the impact of data curation methods, differences in underlying data models can make it more challenging to define exactly equivalent clinical concepts in each database. Researchers should be aware of these potential sources of variability when interpreting results."<br>In my view this is not a fair representation of the Conclusions supported by the study findings and should be corrected accordingly. Scientific communities engaged with health data are working hard towards improving the prerequisites for utilizing existing data sources in contexts different than the original data collection purpose, including improving data quality, its assessment and reporting.<br>Even though the advantages of health data reuse have been largely established, concerns have also been raised with regard to the applicability and reliability of analysis relying on large database pooling (see e.g. Diaz-Garelli F, Johnson TR, Rahbar MH, Bernstam EV, Exploring the Hazards of Scaling Up Clinical Data Analyses: A Drug Side Effect Discovery Case Report, AMIA Jt Summits Transl Sci Proc. 2021; 2021: 180–189, https://www.ncbi.nlm.nih.gov/pmc/articles/PMC8378643/ and Seneviratne MG, Kahn MG, Hernandez-Boussard T. Merging heterogeneous clinical data to enable knowledge discovery https://doi.org/10.1142/9789813279827_0040). A more balanced and critical approach to these methodological solutions and presentation of respective findings is therefore warranted, as has been adopted also in previous similar studies: e.g. Ref. 15 of Gulliford MC et al on antibiotic prescribing : "further work is needed to better understand the quality and completeness of information recorded" in certain areas, which in turn may limit the suitability of utilizing the combined databases in pharmacoepidemiology and pharmacovigilance research.<br>and Ref. 16 Yu D et. al. on "Comparative Estimates in the Annual Consultation Incidence and Prevalence of Low Back Pain and Osteoarthritis in England from 2000 to 2019": in addition to producing conflicting estimates, the authors underlined that these estimates " remain sensitive to analytic decisions and data quality".

**REVIEWER**	Claire Carson<br>University of Oxford, National Perinatal Epidemiology Unit (NPEU)
**REVIEW RETURNED**	22-Aug-2023

**GENERAL COMMENTS**	This interesting manuscript by Dedman and colleagues presents work to explore the feasibility of combining CPRD GOLD and CPRD Aurum datasets. Using both datasets would be very useful, particularly for the study of rare diseases or exposures where maximizing the size of the available population is important. This manuscript is well written and the results are clearly presented. I do have some specific comments / queries about the analyses of overlapping practices and the impact of the Up To Standard (UTS) indicator:

	1. I am not convinced that the comparison of the overlapping practices really addresses the question of consistency between the two datasets for non-overlapping practices - so I'm not sure how useful it is. I may have misunderstood what is being presented here, in which case it needs to be clarified. I think that these are GP practices that were using Vision (GOLD) and moved to EMIS (Aurum), so that the patient data that had been recorded in consultations using Vision are transferred into the EMIS software. This means that what you see when looking at the comparison is a reflection of how well the conversion works (i.e. is what is recorded in EMIS after the practice transferred the data consistent with what had been recorded in GOLD at the time of the consultation?). They map really closely (Fig 1b and 1d), but it doesn't tell you whether the data recorded in EMIS at the time of a consultation would give you the same result.<br><br>2. Similarly, the figures that show the impact of UTS in the overlapping practices have the same issue. What I think we can see here is that the GOLD and Aurum data for the overlapping practices provide a very similar picture (because the EMIS data is a conversion of the original Vision record), and that data is improved when restricted to UTS. The importance of the UTS in the GOLD data is useful to highlight, but it doesn't apply to the main Aurum dataset where data are recorded directly into the EMIS software (and where there is no equivalent indicator of data quality).<br><br>3. While the Aurum may pick up fewer cases than the GOLD (with UTS applied), particularly if you don't augment with the linked data, the fact that the characteristics of the cases are broadly similar in the two datasets is reassuring. Figure 4 is a useful illustration of the differences between the datasets, but the version provided to reviewers is too small to read easily.<br><br>4. The conclusions state that the data are sufficiently similar to combine the datasets, which is fair. However, the results suggest that researchers should perhaps be more cautious when combining data from care prior to 2000. I think it is important to note that differences between the data sources should be explored in any study that chooses to do this - there could be more substantial be discrepancies for other conditions/types of health care utilisation.

VERSION 1 – AUTHOR RESPONSE

RESPONSE TO COMMENTS FROM REVIEWER 1:

The study presents the comparison of baseline characteristics of patient populations in two UK primary databases concerning Huntingdon's disease (HD – a rare condition) and 14 different cancers (10 of the most common types included). The comparison is used as a means of proving feasibility of using data pooled from the two databases, in order to increase statistical power for further studies. The work presented was undertaken in order "to inform a planned investigation into cancer risks among people with HD." (p.6, lines 47-48).

The general topic of large database utilization In observational studies is very topical and even more so when it comes to the more scarcely available and very valuable primary care data, as in the case of this particular study.

My recommendation would be to publish the paper with certain revisions, which could be considered minor, even though in my view they have a significant impact in how the study is perceived and interpreted.

The study itself and its presentation in the manuscript are for the most part rather straightforward, clear and well executed. I did struggle with a few issues though:

• What should the reader expect of the paper based on its title?

• What are the implications of the approach used from the viewpoint of observational study adoption and related requirements?
• In conjunction with the previous question, do the findings presented by the study team support their conclusions?
Starting with the title: I think that the current title, ”Feasibility of pooling patient data from multiple electronic health record IR) databases: Case studies in UK primary care”, to some extent creates too big expectations to the reader and does not really reflect the content of the work presented. I could not understand the use of the term ”case study” and even more so in plural, in the specific context. Moreover, the level of assessing feasibility of pooling patient data based on the approach and findings of the study implies a very specific and (in my view) rather narrow understanding of feasibility.
The comparison undertaken by the study team is indeed the first step in the pathway of utilizing combined patient data, (p5, Introduction -lines 22-23). However, there are still many unanswered questions and additional work ahead, particularly with regard to various aspects of data quality. The aforementioned further steps may eventually render the combination of data either too complex or non-suitable for certain types or whole areas of research questions. Should we in these cases still say that pooling of data is feasible?
I would hence favour a more straightforward approach to the title, providing more specific details of the study content, such as: “Pooling of primary care Electronic Health Record data on HD and cancers: establishing comparability of two large UK databases” or “Combining primary care Electronic Health Record data on HD and cancers: a descriptive study of two large UK databases”.

<Thank you - we agree .The current work does not attempt to provide a definitive answer on the general feasibility of pooling these primary care databases. Our objective is to highlight the need for context-specific assessment of comparability prior to combining patient data from different sources.

We have changed the title in line with the reviewer’s suggestion: “Pooling of primary care Electronic Health Record (EHR) data on Huntington’s disease (HD) and cancer: establishing comparability of two large UK databases”

We have also added a sentence in the introduction to emphasise the specific context and motivation for combining the databases:
“The aim of this study was to establish the feasibility of combining UK primary care data from two CPRD databases, with a focus on examining the capture of Huntington's disease (HD) and cancer in the databases. These are examples of both rare and common diseases, and we also intend to conduct a future study to see whether HD is associated with a lower risk of cancer, which motivated this initial work.”
>>

Moving to the other areas of concern, namely the implications of the study for observational health research studies and the conclusions drawn by the authors.
The authors clearly describe the sources of challenges in combining data from the two databases: Even though the “databases are similar in terms of source population and setting, each database is derived from a different GP software system employing different clinical dictionaries and coding systems, user interface and data capture methods, and data structures.” However, the information provided to the reader with regard to the methodology and the results of code harmonisation work undertaken by the study team is rather limited:
Appendix 1 on page 43 is not really useful for the reviewer/reader since the actually used codings in the two databases are not accessible – only .txt files are visible in the pdf version. This shortcoming does not allow for assessment of the quality of the mappings and choices made by the study team in code harmonisation between the two databases, which is a key issue in EHR-data research use (see e.g. Williams et al. Clinical code set engineering for reusing EHR data for research: A review. http://dx.doi.org/10.1016/j.jbi.2017.04.010).

In addition to more methodological details, also a more specific list of the codelists utilized could be provided in the references instead of the general entry web page of the Data Compass e.g. a) Codelists for: "Risk of fractures in half a million survivors of 20 cancers: a population-based matched cohort study using linked UK electronic health records" or b) Code lists used in "Medium and long-term risks of specific cardiovascular diseases among 108,215 survivors of 20 adult cancers: population-based cohort study using multiple linked UK electronic health records databases" (Accessed and selected as possible examples at: https://datacompass.lshtm.ac.uk/view/types/collection/index.C.html)

<[in Strengths and limitations para2]:
“Migration of EHR data from one clinical system to another in overlapping practices must occur with high fidelity, which is essential to ensure that migrated records continue to support safe and effective clinical management of patients. Under this assumption, overlapping practices provide a setting to assess consistency of codelists and feature extraction algorithms across the two database. The very similar incidence and prevalence of HD, and incidence of specific cancers in overlapping practices therefore provide reassurance that our case definitions were comparable.”
>>

In addition to the limited information provided as to how data quality issues resulting from differences in coding practices and data models underlying the source systems feeding to the two databases were resolved by the study team, the weight of these challenges in regard to combining patient data is in my view underplayed by the authors. The section of Research in content presents a very good overview of all the sources of variability: ”regional differences and their changes overtime, differences in data curation and – most crucially – differences related to the GP software used for documentation: differences in user interface and how this supports recording of accurately coded clinical information, different clinical and drug dictionaries and coding systems, as well as differences in the underlying data models used to represent and store data”
Even though the authors have recognized the sources of problems in data pooling, in the Conclusions section of the paper they skip discussing the impact of these problems on the actual pooling process and analysis phases and only recommend caution in the interpretation of results. “As well as the impact of data curation methods, differences in underlying data models can make it more challenging to define exactly equivalent clinical concepts in each database. Researchers should be aware of these potential sources of variability when interpreting results.”
In my view this is not a fair representation of the Conclusions supported by the study findings and should be corrected accordingly. Scientific communities engaged with health data are working hard towards improving the prerequisites for utilizing existing data sources in contexts different than the original data collection purpose, including improving data quality, its assessment and reporting. Even though the advantages of health data reuse have been largely established, concerns have also been raised with regard to the applicability and reliability of analysis relying on large database pooling (see e.g. Diaz-Garelli F, Johnson TR, Rahbar MH, Bernstam EV, Exploring the Hazards of Scaling Up Clinical Data Analyses: A Drug Side Effect Discovery Case Report, AMIA Jt Summits Transl Sci Proc. 2021; 2021: 180–189, https://www.ncbi.nlm.nih.gov/pmc/articles/PMC8378643/ and Seneviratne MG, Kahn MG, HernandezBoussard T. Merging heterogeneous clinical data to enable knowledge discovery https://doi.org/10.1142/9789813279827_0040). A more balanced and critical approach to these methodological solutions and presentation of respective findings is therefore warranted, as has been adopted also in previous similar studies: e.g. Ref. 15 of Gulliford MC et al on antibiotic prescribing : ”further work is needed to better understand the quality and completeness of information recorded” in certain areas, which in turn may limit the suitability of utilizing the combined databases in pharmacoepidemiology and pharmacovigilance research. and Ref. 16 Yu D et. al. on “Comparative Estimates in the Annual Consultation Incidence and Prevalence of Low Back Pain and Osteoarthritis

in England from 2000 to 2019”: in addition to producing conflicting estimates, the authors underlined that these estimates ” remain sensitive to analytic decisions and data quality”.

<We have amended the penultimate paragraph of the “Research in context” section to give a more balanced summary of previous studies which have noted differences between CPRD GOLD and CPRD Aurum:
“ A second study found that antibiotic prescribing rates for 25 common antibiotics were similar in each database during 2017, particularly when analyses were restricted to England only although the authors recommended further research to understand data quality and completeness around dosage regimes and treatment duration.[15] Another study produced conflicting estimates and temporal trends in incidence and prevalence of low back pain and osteoarthritis between 2005-2019 in CPRD Aurum and CPRD GOLD, and called for further research to understand the impact of analytic decisions and data quality on database heterogeneity.[16] [15][16]Other studies have combined data from CPRD GOLD and CPRD Aurum without presenting direct comparisons between the databases, making it difficult to gauge the potential impact of heteregeneity.”
We have also amended the conclusions section of main the paper and the abstract to to include more qualified statements about whether and when it may be appropriate to combine data:
(Main manuscript) Conclusions:
“Our study focussed on identifying potential sources of heterogeneity in two UK primary care EHR databases, to guide decisions on whether to combine the data for further analysis, and how to properly account for heterogeneity if present.[56,57] The latter involves selection of appropriate statistical models, presenting results of each database separately in addition to pooled results, and design of relevant sensitivity and subgroup analyses. Our comparisons of incidence trends and patient characteristics suggest that we were able to identify similar groups of patients in each database, making it feasible to pool data to increase statistical power for a study of the association between cancer risk in HD patients. Further research is needed to understand observed differences in measures of health care contacts. Differences in HD and cancer incidence trends were seen particularly between 1990 – 2000. This may reflect in part the impact of the UTS data quality filter which is applied to CPRD GOLD but not CPRD Aurum, but further research is needed to understand data quality in CPRD Aurum during this period. Greater caution is therefore warranted when considering whether to combine data prior to 2000. Similar investigations should be undertaken as a preliminary step in any study aiming to combine data from these databases.”
(Abstract) Conclusions:
“Differences in cancer incidence trends between 1990-2000 may relate to use of a practice-level data quality filter (the 'up-to-standard date' [UTS]) in CPRD GOLD only. As well as the impact of data curation methods, differences in underlying data models can make it more challenging to define exactly equivalent clinical concepts in each database. Researchers should be aware of these potential sources of variability when planning combined database studies and interpreting results.”
>>

RESPONSE TO COMMENTS FROM REVIEWER 2
This interesting manuscript by Dedman and colleagues presents work to explore the feasibility of combining CPRD GOLD and CPRD Aurum datasets. Using both datasets would be very useful, particularly for the study of rare diseases or exposures where maximizing the size of the available population is important. This manuscript is well written and the results are clearly presented. I do have some specific comments / queries about the analyses of overlapping practices and the impact of the Up To Standard (UTS) indicator:
1. I am not convinced that the comparison of the overlapping practices really addresses the question of consistency between the two datasets for non-overlapping practices - so I'm not sure how useful it is. I may have misunderstood what is being presented here, in which case it needs to be clarified. I think that these are GP practices that were using Vision (GOLD) and moved to EMIS (Aurum), so that the patient data that had been recorded in consultations using Vision are transferred into the EMIS

software. This means that what you see when looking at the comparison is a reflection of how well the conversion works (i.e. is what is recorded in EMIS after the practice transferred the data consistent with what had been recorded in GOLD at the time of the consultation?). They map really closely (Fig 1b and 1d), but it doesn't tell you whether the data recorded in EMIS at the time of a consultation would give you the same result.

<We have added text in the discussion and in the article summary to make this more explicit….
[in Strengths and limitations para2]:
"Migration of EHR data from one clinical system to another in overlapping practices must occur with high fidelity, which is essential to ensure that migrated records continue to support safe and effective clinical management of patients. Under this assumption, overlapping practices provide a setting to assess consistency of codelists and feature extraction algorithms across the two database. The very similar incidence and prevalence of HD, and incidence of specific cancers in overlapping practices therefore provide reassurance that our case definitions were comparable. Because HD is a rare disease we were also able to perform a simple probabilistic linkage of HD cases from overlapping practices and further demonstrate a high degree of concordance in case identification and classification in each database."
[in Article summary, bullet point 2]:
• we were able to compare data for a subset of the same participants with the same conditions represented in both databases. This provided assurance that case definitions for HD and cancers were comparable in each database.
>>

2. Similarly, the figures that show the impact of UTS in the overlapping practices have the same issue. What I think we can see here is that the GOLD and Aurum data for the overlapping practices provide a very similar picture (because the EMIS data is a conversion of the original Vision record), and that data is improved when restricted to UTS. The importance of the UTS in the GOLD data is useful to highlight, but it doesn't apply to the main Aurum dataset where data are recorded directly into the EMIS software (and where there is no equivalent indicator of data quality).

<
To make this reasoning more explicit we have revised paragraph 7 of the Research in context section:
"Differences in data curation methods – such as availability of a practice level UTS data quality filter in CPRD GOLD but not CPRD Aurum represents a fifth potential source of variability between databases. UTS acts as a filter to remove lower quality data from practices likely to be under-recording, and the effect is to increase incidence rates particularly prior to 2000. The impact of similar data curation measures was reported previously for mortality and incidence of cancer and myocardial infarction in another UK primary care database, THIN, which is also based on data from Vision software.[45,46] However we suspect data quality issues in the early years of computerisation were likely to be widespread and not limited to practices using Vision software. We note also that cancer incidence trends between 1990-2000 for the whole CPRD Aurum database (Figure 2) more closely resemble the trends in overlapping CPRD Aurum practices (Figure 3) where UTS was not applied. Further research is needed to understand the impact of applying a filter similar to UTS date in CPRD Aurum. Researchers should be aware of the potential for differential data quality in the two databases, particularly between 1900-2000, and consider whether it is appropriate to combine data for this period."
>>

3. While the Aurum may pick up fewer cases than the GOLD (with UTS applied), particularly if you don't augment with the linked data, the fact that the characteristics of the cases are broadly similar in the two datasets is reassuring. Figure 4 is a useful illustration of the differences between the datasets, but the version provided to reviewers is too small to read easily.

4. The conclusions state that the data are sufficiently similar to combine the datasets, which is fair. However, the results suggest that researchers should perhaps be more cautious when combining data from care prior to 2000. I think it is important to note that differences between the data sources should be explored in any study that chooses to do this - there could be more substantial be discrepancies for other conditions/types of health care utilisation.

<

We have added specific recommendations in paragraph 7 of the Research in context section:
“Further research is needed to understand the impact of applying a filter similar to UTS date in CPRD Aurum. Researchers should be aware of the potential for differential data quality in the two databases, particularly between 1900-2000, and consider whether it is appropriate to combine data for this period.”

We have amended the conclusions section as follows:
“Our study focussed on identifying potential sources of heterogeneity in two UK primary care EHR databases, to guide decisions on whether to combine the data for further analysis, and how to properly account for heterogeneity if present.[56,57] The latter involves selection of appropriate statistical models, presenting results of each database separately in addition to pooled results, and design of relevant sensitivity and subgroup analyses. Our comparisons of incidence trends and patient characteristics suggest that we were able to identify similar groups of patients in each database, making it feasible to pool data to increase statistical power for a study of the association between cancer risk in HD patients. Further research is needed to understand observed differences in measures of health care contacts. Differences in HD and cancer incidence trends were seen particularly between 1990 – 2000. This may reflect in part the impact of the UTS data quality filter which is applied to CPRD GOLD but not CPRD Aurum, but further research is needed to understand data quality in CPRD Aurum during this period. Greater caution is therefore warranted when considering whether to combine data prior to 2000. Similar investigations should be undertaken as a preliminary step in any study aiming to combine data from these databases.”

We have also amended the conclusions section of the Abstract accordingly:
“Differences in cancer incidence trends between 1990-2000 may relate to use of a practice-level data quality filter (the 'up-to-standard date' [UTS]) in CPRD GOLD only. As well as the impact of data curation methods, differences in underlying data models can make it more challenging to define exactly equivalent clinical concepts in each database. Researchers should be aware of these potential sources of variability when planning combined database studies and interpreting results.”

VERSION 2 – REVIEW

**REVIEWER**	Claire Carson<br>University of Oxford, National Perinatal Epidemiology Unit (NPEU)
**REVIEW RETURNED**	05-Jan-2024

**GENERAL COMMENTS**	The authors have taken on board the concerns or queries raised by both reviewers and have updated their manuscript accordingly. The revised version presents a clearer picture of a carefully conducted and comprehensive exploration of the feasibility of combining the two CPRD databases. Although the focus here is on Huntingdon's disease and a number of common cancers, the principles presented should be considered by researchers working with these data to study other conditions. Therefore, I think it represents a very useful addition to the existing literature. I have no further comments on this and recommend it is accepted for publication.
